# Inclusiveness of social participation for health reform: findings from a realist review

**DOI:** 10.1186/s12939-026-02847-6

**Published:** 2026-05-29

**Authors:** Shraddha Mishra, Neymat Chadha, María Eugenia Esandi, Camila Serrao, Rodrigo Hernan Acuña, Surya Surendran, Amandeep Saini, Ines Aristegui, Devaki Nambiar

**Affiliations:** 1 The George Institute for Global Health, Sydney, Australia; 2 Faculty of Health Sciences, McMaster University, Hamilton, Canada; 3 The George Institute for Global Health, Delhi, India; 4 Research Department, Fundación Huésped, Buenos Aires, Argentina; 5 Faculty of Medicine, University of New South Wales, Sydney, Australia; 6 Prasanna School of Public Health, Manipal Academy of Higher Education, Manipal, Karnataka India

**Keywords:** Realist review, Health reform, Social participation for health, Inclusiveness

## Abstract

**Objective:**

In 2024, the 77th World Health Assembly adopted a resolution on social participation for health (SPH), underscoring the importance of inclusive health system decision-making to achieve universal health coverage. Inclusion-oriented SPH is key to achieving health equity, enabling the needs and perspectives of marginalized communities to reflect in decisions, yet many initiatives fall short. A realist review sought to determine the contextual factors influencing the inclusiveness and outcomes of SPH reform initiatives, alongside their mechanisms of influence.

**Methods:**

Studies were sourced through a comprehensive search focused on social participation, health reform, and inclusion. Following title-abstract screening, quality and relevance were rated using adapted 0–5 scoring. The sub-analysis presented here mapped SPH reform processes, outcomes, geographic spread, and strategies and resources for inclusive SPH.

**Results:**

150 studies were retrieved. Strategies to enhance inclusiveness focused on ensuring access to platforms, tailoring recruitment, and employing culturally safe approaches, and were undergirded by diverse (e.g., financial and technical) resources. 50% of studies reported the nature of diversity in participatory processes, while others addressed qualitative aspects of inclusion/exclusion.

**Conclusions:**

While efforts have enhanced inclusiveness of social participation in health systems decision-making, challenges of meaningful engagement and exclusion remain. There may be no single point or threshold at which complete inclusiveness of social participation for health may be achieved, but it is clear that for populations overall as much as for particular “left behind groups” there are key entry points that may help overcome limitations and barriers.

**Supplementary Information:**

The online version contains supplementary material available at 10.1186/s12939-026-02847-6.

## Introduction

Social participation for health (SPH)—the collective action of social actors toward a common good—encompasses community engagement, citizen participation, and deliberative processes aimed at improving population health [1–3]. SPH is foundational for accountability in achieving primary health care (PHC), is rooted in the legacy of the Alma Ata Declaration, and has been embedded in global movements for health equity since the 1970s [2, 3]. At the 77th World Health Assembly (WHA77) in 2024, Member States endorsed a resolution on social participation for universal health coverage (UHC), health and well-being, calling for inclusive decision-making and emphasizing the involvement of marginalized communities. In so doing, national decision-makers reaffirmed their commitment to developing more equitable and people-centred health systems, promoting access to the full range of quality health services individuals need, when and where they need them, without financial hardship.

SPH can play a crucial role in advancing health equity through the inclusion of marginalized communities. Inclusion-oriented SPH involves purposeful efforts to enhance access to opportunities and resources for participation, particularly for those otherwise excluded. It also involves fostering solidarity among actors, enabling negotiation and redistribution of power [4–6]). The desired outcome of inclusion-oriented SPH is greater inclusiveness, where marginalized groups are equitably involved in participatory processes.

When effectively implemented, SPH can serve as a platform for marginalized communities to voice their needs, address social determinants of health, and promote cultural understandings of well-being [7]. However, many participatory processes, as well-intentioned as they may be, have failed in achieving sufficient inclusiveness, which refers to the extent to which community members are meaningfully included/represented, and on the contrary, have reinforced or perpetuated exclusion and oppression [3, 8–10]. For example, while civil society organizations (e.g., unions) may be included in participatory processes, individuals who are not part of organized groups often remain excluded [8]. Furthermore, logistical challenges, such as the timing and location of participation opportunities, may favour participants with more resources or existing privileges while excluding others. Participatory processes may also lack depth, where community members are involved but not empowered to influence decision-making [9]. In some contexts, they are invited to share experiences but denied decision-making power. Power imbalances and tokenism further undermine the authenticity of participation, leaving community members without meaningful influence [8].

Despite these challenges, promising strategies for broad and deep participation have been documented, such as those that strengthen the SPH capacity of ‘hard-to-reach’ groups [9–11]. However, the documentation and evaluation of these strategies remain piecemeal and largely exploratory [12]. Recognizing this gap, the researchers at Social Participation for Health: Engagement, Research, & Empowerment (SPHERE), a global consortium that integrates research and advocacy to promote social participation for UHC, are conducting a realist review to theorize how the inclusiveness of SPH is enhanced across contexts and communities, and how the nature and extent of inclusiveness shape the broader outcomes of SPH. We focused on health reforms (defined as fundamental changes in the policy and institutional arrangements of the health sector), usually led by governments, to generate evidence for decision-makers and practitioners to use when implementing the WHA77 resolution on social participation and engaging marginalized communities in health reforms [13]. Our focus on health reforms, rather than health more broadly, allows us to balance the scope of the review while aligning with the priorities of SPHERE. The realist review seeks to answer the question: “In what contexts have social participation for health reform initiatives been made more inclusive, by what mechanisms, for which population sub-groups, and with what outcomes?” It aims to highlight mechanisms that can be transferred across contexts to promote inclusiveness in health reforms, exploring how inclusiveness is achieved and why some efforts to achieve inclusiveness fail. The review also examines how varying degrees of inclusiveness impact the outcomes of participatory processes.

This paper presents the initial descriptive and content analysis conducted to group papers for subsequent realist analysis. It summarizes the characteristics of the included studies, the strategies and resources used to enhance the inclusiveness of participatory processes, and the nature and extent of inclusiveness within SPH. We conceptualized strategies as broad approaches for enhancing the inclusiveness of SPH, while activities were defined as more concrete efforts undertaken as part of the strategies [14]. Given the strong thematic overlap between the strategies and activities identified, we integrated these categories in our analysis. Resources were defined as materials, staff, funding, and other assets available and/or used to enhance the inclusiveness of SPH [14].

## Methodology

This manuscript reports on a sub-analysis of papers shortlisted as part of a realist review of SPH.

The parent review followed a five-stage process, with synthesis design adhering to the realist review framework established by Pawson et al., [15] and the Realist and Meta-narrative Evidence Synthesis: Evolving Standards (RAMESES) guidelines [15, 16]. The five stages are summarised in Supplementary File A; the protocol was prospectively registered in PROSPERO (CRD42023440695), with the caveat that realist syntheses support iteration in their design.

For the analysis reported within this manuscript, we indexed strategies, activities, and resources for (inclusiveness in) SPH, alongside the nature and extent of inclusiveness achieved, in preparation for subsequent (realist) analyses [17]. To achieve this, we used hybrid content analysis combining deductive and inductive approaches [18]. Deductively, our coding was guided by concepts drawn from the research question and the realist orientation of the review [15, 16] which provided an initial structure for organising extracted material. Inductively, we allowed patterns within the data to shape and refine these categories through constant comparison, an approach consistent with established qualitative analytic traditions [19, 20].

To begin, all extracted data were exported from Covidence as a .CSV file. One author then immersed in the data extractions in order to propose descriptive codes eventually collated within a codebook. Codes regarding the nature and extent of inclusiveness achieved were deductively developed, consisting of the three dimensions of inclusiveness (numeric diversity, meaningful participation, and experiences of inclusion or exclusion) that we had conceptualized based on existing literature [21–23]. They were supplemented with the inductive codes pertaining to successes and challenges around inclusion. Meanwhile, codes categorizing strategies, activities, and resources for (inclusion in) SPH were inductively developed based on the extracted data [14]. Indexing was reviewed and refined during weekly team meetings. Once a comprehensive set of codes had been developed and refined, one reviewer examined the extractions for each paper, identifying the presence of the codes, though each code was verified – and, if warranted, adjusted – by a senior reviewer. A dual verification approach was used to enhance the credibility of the analysis. Codes were ultimately grouped by inductively gleaned themes.

Complementing the coding process, analyses of strategies and resources for particular population subgroups were carried out and reviewed, alongside strategies that applied across groups. We included an analytic step in which each extracted strategy, activity, and resource was tagged with population‑subgroup identifiers (e.g., Indigenous communities, racialised groups, migrants, people with disabilities, gender‑diverse populations). These subgroup‑specific datasets were then compared to identify mechanisms, contextual influences, and recurring patterns unique to (or shared across) subgroups, and coding decisions were cross‑checked by at least two team members. Strategies that applied across groups were reviewed in parallel.

Recurring features across the extracted material were placed into four cross‑cutting dimensions that subsequently structured the Results: who is able to participate; how opportunities for participation are arranged and accessed; whether participatory spaces are experienced as culturally and socially safe; and the extent to which participants feel prepared to engage. In parallel, repeated descriptions of how participation was characterised in the included studies informed the identification of three aspects of inclusiveness: the diversity of participants involved, the opportunities available for meaningful contribution, and participants’ reported experiences of inclusion or exclusion. These dimensions and aspects emerged through progressive comparison, refinement, and consensus among the review team. Future analyses will extend this work through the development of context-mechanism-outcome configurations for the substantive SPH ‘types’ identified in the current analysis.

## Results

The Results are organised thematically to reflect patterns identified through the combined deductive–inductive analysis. Four dimensions consistently shaped the inclusiveness of SPH—who is able to participate, how opportunities are organised and accessed, whether spaces are experienced as culturally and socially safe, and the extent to which participants feel prepared to engage. In addition, inclusiveness was described across three interconnected aspects: the diversity of participants present, opportunities to contribute to discussions or decisions, and reported experiences of inclusion or exclusion. The subsections that follow present these patterns alongside illustrative examples and note contextual variations observed across settings.

### Study sample

A total of 150 papers possessing adequate quality and discussing inclusiveness for SPH were retrieved. Of the 150 papers, 125 examined SPH in a single country and 16 in multiple countries (see Supplementary File A). The greatest number of single-country studies were in the US (*n* = 30) followed by Colombia (*n* = 11), South Africa (*n* = 11), Canada (*n* = 9), Ghana (*n* = 9), India (*n* = 9), Australia (*n* = 8), Tanzania (*n* = 8) and United Kingdom (*n* = 8). Among these, eight could not be assigned to any specific country as they were either multi-country in scope or lacked a distinct country focus [24–31]. Across the papers reviewed, all global regions except northern and western Asia were represented.

The indexed strategies, activities, and resources for inclusion in SPH, alongside the nature and extent of inclusiveness achieved, are summarized (Tables 1 and 2) and discussed below.

**Table 1:** Indexed strategies, activities and resources for inclusion in SPH

Codes pertaining to strategies & activities for inclusion	Total articles	Codes pertaining to resources for inclusion	Total articles
***Overall inclusion*** Promoting inclusion overall rather than inclusion of specific groups, e.g., participatory mechanism which has an overarching focus on equitable representation without defining specific groups	*n* = 37	***Financial resources*** Financial resources which support inclusion	*n* = 14
***Inclusion of specific groups*** Promoting the inclusion of specific groups, e.g., focus on better involving youth in participatory processes	*n* = 58	***Human resources*** Human resources which support inclusion, e.g., healthcare providers, CHWs	*n* = 23
***Solidarity*** Solidarity across topic/issue areas e.g., strategizing to be able to gain entry into a coalition as an organization	*n* = 11	***Technical resources*** Evidence, datasets, data collection tools, reports; technical skills	*n* = 9
***Inclusive language*** Using language which eases participation for different groups, e.g., using Indigenous languages, ensuring that all participants understand their roles to promote their participation	*n* = 4	***Incentives or compensation*** Non-monetary or monetary incentives which different subgroups get for participating, e.g., access to free healthcare	*n* = 3
***Training*** Anything to strengthen the capacity of actors with the aim of enhancing inclusion. e.g., training of marginalized community members in user rights and important skills for participation, improving government capacity to involve minoritized groups	*n* = 29	***Legislation or policy*** Any law or policy which promotes inclusive participation, e.g., a national policy document which encourages the participation of a group	*n* = 9
***Feedback and monitoring*** Monitoring of how inclusive spaces are and what barriers are faced with report directly from community members	*n* = 8	***Political support*** Political support for inclusive participation	*n* = 10
***Cultural safety / Intercultural approaches*** Respecting community cultures and norms, e.g., in the design of educational materials	*n* = 14	***Technological resources*** Medicines, essential supplies, digital technology & other material innovations for inclusive participation, e.g., social media allowing outreach to young people	*n* = 5
***Leveraging relationships*** Capitalizing on relationships to promote inclusion, e.g., using our relationships with non-governmental organizations since they can bridge us with marginalized community members	*n* = 19	***Community resources*** Resource(s) provided by community to promote inclusion, e.g., land donated by community	*n* = 3
***Strategic recruitment*** Recruitment of participants in a way that promotes inclusion, e.g., extending face-to-face invitations to participate to marginalized groups, targeted outreach to specific groups, selection of representatives by community leaders to ensure diversity	*n* = 22	***Community assets*** Strengths that exist in a community to promote inclusion, e.g., Principle of ulinganya (equality of treatment of different people) exists in a community	*n* = 12
***Diversity quotas*** Requiring the participation of certain subgroups, e.g., legal mandates for the participation of certain minoritized groups	*n* = 8	***Insufficient resources*** Instances where there haven’t been enough resources for inclusion, e.g., insufficient support from government actors created role confusion which ultimately lessened participation of certain groups	*n* = 16
***Inclusive facilitation*** Facilitating dialogues in a way that promotes inclusion, e.g., promoting non-discrimination, tailoring meeting structure to the needs of various groups, conflict management	*n* = 13		
***Mechanisms for participation*** Choosing mechanism(s) of participation which are accessible, e.g., protected space for a single community, allowing feedback to come in from outside official channels	*n* = 30		
***Philosophy or ethos*** Having inclusion as an overall guiding principle, e.g., ulinganya (equality of treatment of different people) and ukushikwete akapatulula (impartiality when tackling issues)	*n* = 18		

**Table 2:** Summary of findings regarding the nature and extent of inclusiveness achieved

Codes pertaining to numeric diversity	Total articles	Codes pertaining to meaningful participation	Total articles	Codes pertaining to experiences of inclusion or exclusion	Total articles
***Numeric diversity (Mention)*** Article describes groups which gain or do not gain entry into participatory process and/or articles states that there is or isn’t a diversity of groups present	*n* = 81	***Meaningful participation*** Article describes whether different subgroups are participating fairly (e.g., they are able to speak freely)	*n* = 65	***Experiences of inclusion or exclusion*** Article describes how different subgroups feel included or excluded (e.g., people of low socioeconomic status report feeling like they are not taken seriously)	*n* = 50
***Numeric diversity - Successes***	*n* = 48	***Meaningful participation - Successes***	*n* = 29	***Experiences of inclusion or exclusion - Successes***	*n* = 11
***Numeric diversity - Challenges***	*n* = 52	***Meaningful participation - Challenges***	*n* = 43	***Experiences of inclusion or exclusion - Challenges***	*n* = 43

### Efforts to enhance the inclusiveness of SPH

Across the reviewed studies, four broad dimensions appeared to shape the inclusiveness of SPH processes: who was able to participate; how opportunities were arranged and accessed; the extent to which spaces were experienced as culturally and socially safe; and the degree to which participants felt prepared to engage. These dimensions arose inductively during analysis and were observed across a range of contexts; the sections that follow describe strategies and resources aligned with each dimension.

#### Strategies and activities for inclusion

**Fig. 1:**
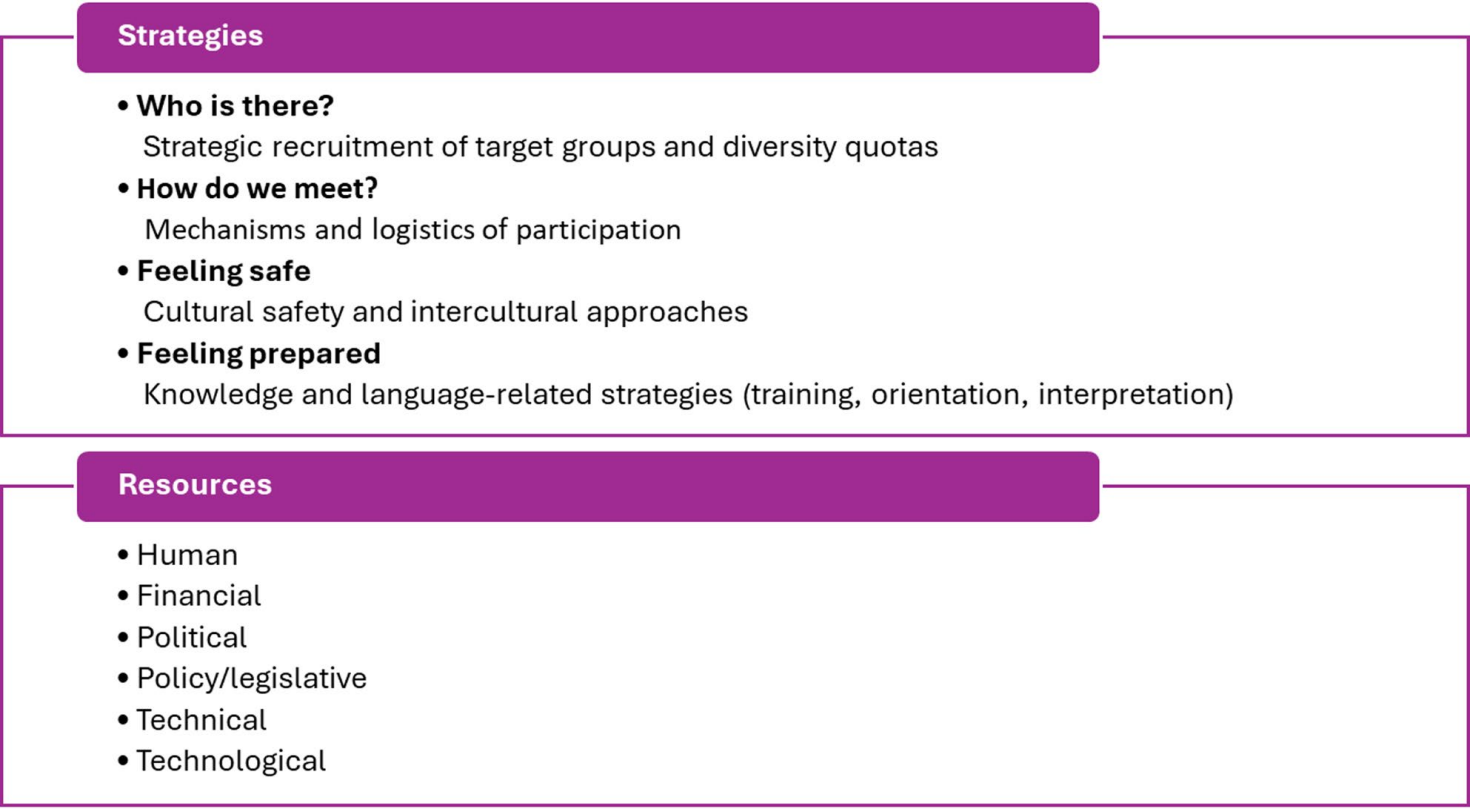
Summary of strategies and resources for inclusive SPH

Efforts for inclusive SPH were seen in a large number of countries, and were focused on a range of groups, such as Indigenous (including First Nations) communities, youth, ethnic minorities, migrant communities, Lesbian, Gay, Bisexual, Transgender, Queer, Intersex (LGBTQI+) people, persons living with disabilities, health workers, persons who use drugs, cultural and religious leaders, and groups at specific intersections such as women who are mothers, youth who are street involved, and older adults living with chronic disease. Box A outlines the specific strategies for inclusion of these groups in SPH and in the following sections, we summarise strategies as well as resources reported in the literature to enhance inclusiveness (see Fig. 1).

#### Who is there?

**Strategic recruitment of target groups** to ensure their representation on community action platforms was also observed (*n* = 22). In Zambia, the recruitment of older women, men, and traditional birth attendants, who significantly influence pregnant women’s health-related behaviours, was intentional in efforts to initiate community engagement and ensure pregnant women made health related behaviour changes [32]. In the Netherlands, face-to-face invitations, complemented by informational sessions on user rights, were utilized to engage individuals in mental health advocacy [33]. In the US, to ensure that African American middle school students’ representation in community violence prevention efforts would be sustained in case of a decline in participation, they were invited in relatively high numbers [34].

In some country contexts, inclusiveness was ensured through **diversity quotas** (*n* = 8). In Scotland, a Social Inclusion Partnership mandated representation of community members on its governing board, including a designated position for a youth member [35]. In Kenya, county assembly membership was subject to gender composition quotas, requiring representation of persons with disabilities, and including affirmative action measures to promote diverse participation [36]. In Brazil, health councils—established under Federal Law No. 8142/1990 and tasked with participatory health planning and reform—were mandated to include medical associations, users’ associations, and unions, thereby fostering a broad spectrum of community engagement [37].

#### How do we meet?

The strategies observed were intended to enhance inclusiveness and many of them were related to the logistics of participation. Introducing or adapting **mechanisms of participation** to facilitate broad-based involvement was among the most cited (*n* = 30). This included scheduling meetings at times that allowed young people to participate in USA [38], allowing feedback submissions outside of structured consultations in the UK [39], holding dialogues and events in accessible locations, such as school buildings, places of worship and recreational centres in Uganda [40], remunerating those who would lose income for participating on working days in Nigeria [41], and creating spaces such as popular assemblies, community meetings, and community management circles for decision-making [42].

#### Feeling safe

**Cultural safety and intercultural approaches** were also commonly employed to ensure inclusion. Examples included efforts to centre Indigenous and First Nations cosmologies in the mobilization for primary health care reform in Canada [43], a practice noted as absent in a study examining Aboriginal Health Worker experiences in Australia [44], and engagement of church leadership for HIV-related mobilization in Uganda to address and resolve dissonances between cultural and religious beliefs and recommended HIV practices [31]. In Argentina, substance use has been addressed through the empowerment of former users to facilitate outreach, and by building trust with health professionals through daily interactions [45].

#### Feeling prepared

A great number of papers (*n* = 29) reported the use of **knowledge-related strategies** intended to strengthen the capacity of actors and, by extension, enhance inclusiveness. In India, the Self Employed Women’s Association coordinated leadership training and information dissemination among community members (e.g., about their health service entitlements) to promote their involvement [46]. In Timor-Leste, meanwhile, community members involved in mental health policymaking received training in human rights language and principles from disability rights organizations [47]. In Colombia, there was a training cascade in which community leaders received training and subsequently imparted it to women with high levels of vulnerability, aiming to improve their knowledge about cervical cancer and their rights to access health services [48].

We also saw a focus on **accessible language**, specifically ensuring Indigenous peoples’ languages, such as Ojibwe and Dakota, were included in educational materials in the US [49] and the engagement of translators in Australia [50] and Spain [51] (*n* = 4).

#### Resources for inclusion

A range of resources (used for) facilitating inclusive SPH were observed, including **human resources** (*n* = 23), **financial resources** (*n* = 14), **political support** (*n* = 10), **the support of legislation or policy** (*n* = 9), **technical resources** (*n* = 9), **technological resources** (*n* = 5). **Notable human resources** included facilitators whose knowledge of community histories fostered a sense of security within dialogues [39] designated health volunteers tasked with facilitating the participation of men and women respectively [52], and professional organizers tasked with the management of participatory processes [53], with authors noting that the presence of substantial human resources facilitated broad-based governance [8]. **Financial resources** include government subsidies and funding from non-state institutions, which—in the USA—enabled a wider range of community organizations to participate in a health collaborative [54], and helped compensate participating community members [55]. In terms of **political support** for inclusive SPH, Aboriginal Community-Controlled Health Services (ACCHSs) in Australia were noted to have amplified the health concerns of Indigenous communities [12]; in Timor-Leste, a broader government focus on the social inclusion of people with mental health challenges facilitated their participation [47]. Highlighted examples of **supportive legislation and policy** include the constitution-mandated participation of communities experiencing vulnerability in Uganda [31], pre-existing structures intended to facilitate the participation of people living with disabilities in Timor-Leste [47], and the 94 Calls to Action by the Truth and Reconciliation Commission of Canada, intended to address the impacts of colonialism on Indigenous peoples [43]. Observed **technical resources** included capacity-strengthening tools and activities aimed at actors less familiar with policy-related processes. In Mali [56], these ranged from self-care and dialogue with caregivers to peer education and policy advocacy, while in the USA [54], they included team-building exercises and email campaigns. With respect to **technological resources**, the use of multimedia supported awareness-raising among community members. Graphics used in Argentina, for instance, enhanced the process of comprehension [57]. In Guatemala and India, communications technology platforms enabled anonymous reporting by community members, making participation in efforts to strengthen health services more accessible [58].

### Nature and extent of inclusiveness

Eighty-one studies reported the extent of numeric diversity within participatory processes, 65 described the degree to which people experiencing marginalization were meaningfully involved, and 50 noted experiences of inclusion and exclusion.

#### Successes related to inclusion

Forty-eight studies noted the achievement of numeric representation of one or more groups, 29 discussed the meaningful participation of one or more groups, and 11 described experiences of inclusion.

Examples of numeric representation included the grassroots mobilization of “Black, unemployed, poorly educated, young HIV-positive women from poor communities” in advocacy around HIV/AIDS crisis in South Africa [59], representation from Dalit community members in the majority of Health Facility Operation and Management Committees in Nepal [60] and diverse community representation in the development of local mental health services [61].

Meaningful participation by people experiencing marginalization was exemplified in Canada, where certain Indigenous communities were described as having achieved control over their health data [43]; in Malawi, where traditional chiefs facilitated the resolution of disputes between the community and health care providers [62]; and in the Netherlands, where a participatory initiative gave people living with mental illness the opportunity to exchange lived experiences [33].

Firsthand experiences of inclusion were reported in Australia, where the separation of Rohingya men and women during community-led events fostered connectedness between women, and the employment of Aboriginal and Torres Strait Islander Health Workers (AHWs) within Aboriginal community-controlled health services cultivated positive experiences for AHWs [44, 63]. In Colombia, health system user associations, through the possibility of being heard and communicating their needs, as well as through tactics aimed at neutralizing power asymmetries, enabled a sense that participation is a pathway to recognition and inclusiveness [64].

#### Challenges related to inclusion

A significant portion of the evidence highlighted the underrepresentation of groups critical for SPH (*n* = 52), inadequate or tokenistic participation of groups (*n* = 43), or outright experiences of exclusion (*n* = 43).

Studies (*n* = 52) noted the underrepresentation of community members marginalized on the basis of age, caste, class, disability, gender, illness, immigration status, language location of residence, and/or race, and of health service users, traditional healers, and Indigenous peoples. One study described members of elite groups as less likely to engage in SPH if they perceived service decisions as unlikely to impact them, highlighting that the absence of solidarity can hinder the outcomes of SPH [41].

The quality of marginalized groups’ participation was also seen to be undermined by power asymmetries, denying certain communities decision-making power and preventing them from being heard. For example, in the USA, women’s health movements were dominated by white women, excluding the perspectives and needs of racialized women [65]. In many participatory processes in high-income countries, final decisions were made by individuals in professional leadership roles [28]. Additionally, a study focused on Benin, Kenya, and Zambia reported that women were excluded from leadership roles [66].

Accounts of exclusion included reports of stigma and discrimination along various dimensions of (in)equity (e.g., racism and casteism). In Nigeria, low-income community members expressed concerns that they would not be taken seriously given stigmatizing beliefs that they are uneducated [41]. In the USA, Black women described overt sexism and paternalism within Black health movements [65], and in India, the pervasiveness of caste discrimination found its way even in SPH platforms. One Dalit participant, facing marginalization on the basis of caste, described their experience in an SPH committee as:

*In the committee*,* most of the members are from higher castes. When we have meetings of the committee or any other program*,* and when there is time for taking snacks*,* the other committee members sit a short distance away from me. There is thus still discrimination in our society* [60].

Notably, 16 studies reported an insufficiency of financial and other resources to ensure the inclusiveness of SPH. In Timor-Leste, for example, a lack of resources made it challenging to bring mental health services users together to participate in policy making [47].

#### Box A. Inclusion-focused platforms for social participation

*We saw instances of an expressed focus on inclusion in social participation for health that were thematic*,* population-focused or related to governance. Globally*,* we found that mobilisation and organisation related to HIV/AIDS was a major catalyst for participation of “key populations” and groups that faced “marginalisation.” A great emphasis of these efforts was addressing stigma and overcoming functional and foregone care of these communities* ([67, 68].

*There were some population-level variations as well. In North America*,* the US in particular*,* many examples of community based participatory research were found that focus on inclusion of racial and ethnic groups experiencing marginalization* [54, 69, 70]. *In other High Income Countries*—*not just in North America*,* but also in other regions of the world*—*young people have been involved in platforms for multisectoral action focused on food*,* neighbourhood safety and violence prevention* [34, 35, 71]. *Across regions*,* but in particular in Africa*,* the Americas and Oceania* [43, 72], *we saw concerted efforts involving First Nations and Indigenous populations—these were focused on health rights and tackling the historical legacies of exploitation that have affected the health status of these communities. In a number of countries*,* like Uganda* [31], *Kenya* [36], *India and Brazil decentralisation reforms have been used as a platform for health and have served as a base for enhancing inclusiveness in decision-making.*

## Discussion

The themes identified in the Results, including who participates, how participatory processes are organised, and the conditions shaping participants’ experiences, provide a foundation for interpreting the wider implications of SPH initiatives' broader patterns across the reviewed literature. Building on these themes, this sub‑analysis of our broader realist review offers insight into how SPH can be meaningfully integrated into health system reforms. As a descriptive, preparatory stage of the larger realist review, this sub‑analysis identifies patterns that will inform but do not yet constitute the development of full context–mechanism–outcome configurations.

Our findings from this sub-analysis advance the understanding of how SPH can be meaningfully integrated into health system reforms. By synthesizing evidence from diverse global experiences, it identifies key strategies and enabling resources used to engage structurally excluded groups through strategic recruitment, capacity-building, and culturally safe participatory mechanisms.

While some initiatives demonstrated meaningful inclusion and influence in decision-making, the persistence of symbolic participation highlights ongoing barriers to genuine power-sharing. These findings offer critical insights into the conditions under which SPH can move beyond tokenism and contribute to more equitable and inclusive health governance. As reflected in the Results, patterns relating to representativeness, logistical access, cultural safety, and preparedness help explain why some efforts achieved depth while others remained largely symbolic. Framing these findings through a realist lens highlights that inclusiveness is not simply the result of a single strategy but emerges when specific mechanisms are triggered in particular contexts. Our mapping suggests that empowerment-oriented mechanisms are particularly potent when combined with enabling.

Overall, our review reveals a modest literature base referencing inclusiveness of SPH globally. Examples spanned the globe although, yet we found that northern and western Asia as well as the Mediterranean and Middle East regions appeared to be under-represented. While some countries emphasize formal structures (e.g., Brazil’s local health councils) [37], others rely on informal networks and grassroots mobilization [73]. Although these efforts demonstrate a commitment to embedding equity in health governance, critical challenges persist. Particularly, we found that inadequate or tokenistic participation of population groups is prevalent in SPH efforts, along with feelings of exclusion from community members. Morgan, [2] cautions that participation often serves as a political symbol, co-opted by institutions to serve varied agendas [2]. This tension is evident in our review, where policy mandates seek to institutionalize participation, yet resource constraints and tokenistic practices persist [31, 43]. In several contexts, limited funding hindered the ability to convene marginalized groups, thereby undermining the inclusiveness of participatory processes [54, 74, 75]. Power asymmetries, particularly in high-income countries, often relegated community members to advisory roles without decision-making authority [76]. The dominance of elite or professional actors in final decisions diluted the transformative potential of SPH [77, 78]. Despite the mobilization of resources and strategic efforts, our findings echo Morgan’s [2] assertion that participation is inherently shaped by power dynamics [2]. Without addressing systemic disempowerment, particularly among historically marginalized groups, the transformative potential of inclusive SPH remains limited. The WHO Handbook similarly warns that without clear mandates, transparent selection processes, and feedback mechanisms, participatory spaces risk being perceived as symbolic rather than substantive [3]. As evident in our sub-analysis, these challenges were observed alongside reports of under‑representation, limited influence, and experiences of discrimination, indicating that structural and relational barriers frequently co‑occur.

Strategies to enhance the inclusion of marginalized groups in SPH processes were highly diverse and context specific. However, our sub-analysis identified four cross-cutting elements that helped categorize these efforts: how individuals were recruited, how their active participation was supported, and how they were made to feel both prepared and safe to engage [31–37, 39–41, 43, 44, 46–49]. This underscores the importance of attending not only to the “who” and “what” of participation, but also to the “how” and “under what conditions,” particularly when aiming to include those historically excluded. A core focus on mechanisms of participation resonates with the WHO Handbook on Social Participation for Universal Health Coverage which emphasizes the importance of designing participatory spaces that actively counteract power asymmetries and promote civic empowerment [3]. The handbook provides a comprehensive typology of participatory mechanisms and highlights the need for capacity-building—both among community actors and within government institutions—to ensure equitable engagement. These insights align with our analytical emphasis on structural inclusion and an empowerment model of participation. Morgan [2] contrasts such an empowerment based approach with the Utilitarian Model, which views participation as a means to improve efficiency [2]. Strategies for inclusiveness seen in our review, affirmative action measures, intentionality, cultural sensitivity, and structural inclusion, appear to align more with an empowerment-based approach.

Our findings align with insights from Pagatpatan’s realist review, which highlights the importance of tailoring public participation processes to reach marginalized or “hard-to-reach” groups through techniques such as separate deliberations, digital tools, and multi-method approaches [79]. While that review emphasizes structural and procedural mechanisms to promote inclusiveness, our findings further stress the relational and emotional conditions—such as feeling safe and prepared—that enable meaningful engagements; and where cultural safety and capability strengthening were often necessary precursors to active contribution.

Across diverse contexts, resources were not only mobilized to support service delivery but also to strengthen participatory structures and processes. In Kenya and Cambodia, for example, community representatives were directly involved in fund management, and revenues from user fees were used to compensate health facility committee members—highlighting how financial incentives can reinforce accountability and engagement [25, 78]. Similarly, in Uganda and Zambia, material and financial support, such as bicycles for community health workers were instrumental in sustaining community-driven initiatives [32, 80]. Institutional mechanisms also played a key role: India’s state-level innovations and Malawi’s use of legal frameworks to address health worker misconduct illustrate how governance structures can be leveraged to support decentralized, participatory action [62, 73, 81]. Moreover, countries like Sweden and South Africa demonstrated how national strategies and training programs can institutionalize collaboration between municipalities, health workers, and communities [61, 82]; thus collectively suggesting that participation is most effective when it is not only invited but also resourced and supported through formal mechanisms.

The NHA’s evolution from a complaint platform to a solution-oriented forum reflects a shift toward civic empowerment [83]. This mirrors broader trends identified in our sub-analysis, where knowledge-related strategies, such as leadership training and rights education, empower participants and democratize access to health governance [46–48]. These efforts are particularly important in contexts where historical marginalization has eroded trust in formal health systems [24]. By equipping community members with the tools to understand and navigate health systems, these initiatives transform passive recipients of care into active agents of change [84]. The cascade training model in Colombia is a notable example of how capacity-building can be scaled and sustained within vulnerable populations [48]. These findings from our sub-analysis echo Marston et al., [85] focus, which identifies three interdependent areas for transformative participation for inclusive and sustainable participation: (i) building individual and community capabilities; (ii) developing people-centred health services; and (iii) strengthening social accountability mechanisms [85]. Taken together, these strands of evidence illuminate the foundational patterns that the next stage of the broader realist review will interrogate more deeply through context–mechanism–outcome analysis, providing an empirical map for developing nuanced context-mechanism-outcome configurations.

### Strengths

This review has some key notable strengths. First, it includes literature in both English and Spanish, allowing for broader geographic and cultural representation, particularly from Latin America, which is often underrepresented in global health research. Second, our comprehensive search strategy was designed to capture a wide range of literature on inclusion in SPH, including peer-reviewed articles and grey literature, enhancing the depth and diversity of our evidence base. Third, we employed a rigorous data collection process, with dual coding and review of extractions by a second team member. Throughout the study selection (screening) process, especially where dual screening was not possible, the team consistently met to build shared understanding of the underlying concepts, including through calibration ‘tests’ developed by the first author. This collaborative approach, focused on balancing sensitivity and specificity of the screening and extraction processes, helped ensure consistency, reduce bias, and strengthen the reliability of our findings.

### Limitations

One key challenge was the complexity and ambiguity of terminology, particularly around concepts such as “inclusiveness” and “meaningful participation.” These terms were variably defined across the literature, which created difficulties during screening, extraction, and coding with multiple team members involved at various stages of the process. As a result, some decisions about what ‘counted’ as inclusiveness may have been inconsistent. Additionally, some stages of the review involved single screening, which may have affected the sensitivity and specificity of our data collection. While we attempted to mitigate this through team discussions and spot checks, it remains a limitation that could have influenced the comprehensiveness of our findings. Finally, due to the rigour criteria, some articles showcasing relevant exemplars of SPH may have been excluded because they did not meet the cut-off scores set for appraisal.

### Implications for research

Our findings underscore that achieving inclusive and transformative change requires a myriad of strategies and a diverse range of resources, reflecting the complexity and contextual variability of such efforts. Despite numerous initiatives, existing approaches often fall short of sustaining meaningful, long-term transformation, highlighting the dynamic and evolving nature of inclusion work. This suggests an imperative for academia to adopt more adaptive, fit-for-purpose methodologies. In particular, longitudinal studies employing ethnographic or other flexible methods may be essential to capture how SPH initiatives function, adapt, or falter over time.

### Implications for policy

From a policy perspective, the literature analysed as a part of our review revealed several key entry points for enhancing inclusiveness within social participation for health, making a strong case for the benefits of expanding inclusive practices. However, policy must also recognize that this is a dynamic and evolving space—solutions that were effective in the past may not necessarily address the complexities of future programming. At the same time, the legacies and historical trajectories of SPH initiatives, including their successes, challenges, and discontinuations, offer valuable lessons. These insights can inform more adaptive, context-sensitive policy frameworks that are better equipped to support sustainable and inclusive SPH programming moving forward.

More broadly, our findings may be transferable spaces and processes beyond SPH, and in particular for actors seeking to enhance inclusiveness. We reinforced that inclusiveness is a multidimensional construct, encompassing not only the presence of groups but also their meaningful participation and sense of belonging within processes, demanding attention to both numeric diversity and inclusion. Moreover, while the people establishing processes and spaces such as SPH may be best placed to facilitate inclusion (e.g., through proper resourcing), a collective effort by all those involved seems important, seeing as participating community members in addition to SPH implementers influenced the extent of inclusiveness. Lastly, in line with various critical theories (e.g., intersectionality), the nature and success of inclusion seem context-bound, requiring implementers to work with the strengths and challenges specific to their sociopolitical, geographic, and other contexts [86].

## Conclusion and next steps

This paper presents a sub-analysis of our realist review exploring the contextual factors influencing the inclusiveness and outcomes of SPH reform initiatives, alongside their mechanisms of influence. Strategies to enhance inclusiveness focused on ensuring access to platforms, tailoring recruitment, and employing culturally safe approaches, and were undergirded by diverse (e.g., financial and technical) resources. While many of the studies described successful efforts to promote the inclusiveness of SPH initiatives, much work remains to address ongoing challenges. The next phase of our study, involving context-mechanism-outcome (realist) analysis stratified by key characteristics of SPH initiatives, will provide context-sensitive guidance for promoting inclusiveness.

## Supplementary Information

Below is the link to the electronic supplementary material.

Supplementary Material 1

## Data Availability

The studies included within this review are publicly available online. Their characteristics have been summarized in the supplementary information file.

## Abbreviations

SPH: Social participation for health
WHA77: 77th World Health Assembly
CMO configurations: Context–mechanism–outcome configurations
LGBTQI+ people: Lesbian, Gay, Bisexual, Transgender, Queer, Intersex+
