# Supplementary material for: Inclusiveness of social participation for health reform: findings from a realist review
Source: Int J Equity Health. 2026 May 29;25:171. doi: 10.1186/s12939-026-02847-6 (PMC13397718; doi:10.1186/s12939-026-02847-6)
Supplement: Supplementary file 1 — Supplementary Material 1 [file 12939_2026_2847_MOESM1_ESM.docx]

# Supplementary File A

# This supplementary file summarizes the methodology being used within this realist review alongside the characteristics of included studies.

#

# Methodology

The realist review is following a five-stage process, with synthesis design adhering to the realist review framework established by Pawson et al. (2005) and the Realist and Meta-narrative Evidence Synthesis: Evolving Standards (RAMESES) guidelines (Pawson et al. 2005; Wong et al. 2013). The five stages included: (1) developing and refining the research question; (2) conducting evidence searches and selecting relevant studies; (3) appraising studies and extracting data; (4) synthesizing evidence and drawing conclusions; and (5) disseminating findings. Stages one to four are described below. The protocol was prospectively registered in PROSPERO (CRD42023440695), with the caveat that realist syntheses support iteration in their design.

**1. Development and refinement of research question**

The research question was developed collaboratively by the research team, SPHERE members and realist review methodology experts. A preliminary review of existing realist syntheses on SPH demonstrated that each SPH activity is characterized by distinct contexts, mechanisms, and outcomes, making it impractical to address all activities comprehensively within a single synthesis. Guided by these findings, established realist review practices, and SPHERE’s strategic priorities, the scope was refined to focus specifically on the context-mechanism-outcome configurations of SPH activities that are shaping and shaped by social inclusiveness. In-depth review of the included texts revealed substantial variability in the contexts they examined, necessitating multiple realist analyses, with each focused on a distinct intervention context. Therefore, descriptive and content analyses, which could aid in the classification of study contexts, were necessary, and the findings of these analyses are presented in this paper.

**2. Evidence search and selection**

The search strategy was developed in collaboration with a specialist research librarian.

Searches were conducted in PubMed, Embase (Ovid), CINAHL (Ebsco), Web of Science, ProQuest Theses and Dissertations, PDQ, and the Virtual Health Library (BVS) from mid- to end-2023. Supplementarily, the first five pages of Google Scholar results were searched. The search strategy was organized around three thematic blocks: social participation, health reform, and inclusion/inclusiveness (health equity). Searches were performed in both English and Spanish, employing Medical Subject Headings (MeSH), Descriptors in Health Science (DeCS), and free-text terms for each block. Relevant and specific terms were identified by systematically reviewing definitions, subheadings, and categories provided by the MeSH and DeCS databases. The full search strategy can be found in the section of this document titled “Search strategy””.

An iterative process of preliminary searches was conducted to evaluate the breadth and volume of results. Search results were exported to and screened in Covidence, which automatically removed duplicates. The chosen inclusion and exclusion criteria were guided by the Preferred Reporting Items for Systematic Reviews and Meta-Analyses (PRISMA) guidelines. The research team iteratively refined the scope and relevance of each criterion in discussions throughout the screening process (Table 1). Notable deviations from our published protocol include the exclusion of French papers, grey literature, and theses/dissertations, given the volume of eligible papers exceeding team capacity.

**Table 1. Inclusion and exclusion criteria**

| **Category** | **Inclusion Criteria** | **Exclusion Criteria** |
| --- | --- | --- |
| **Population** | Includes information on participation of the public or specified population sub-group (defined by geography, social or economic identity/ characteristics, health condition) or community members of a specific country or specific countries | Includes information only on involvement/role of (technical) experts, health professionals, or decision-makers in health reform/policy making |
| **Intervention** | Describes social participation for health reform and/or strategies, activities, and resources proposed to enhance inclusiveness of participatory processes for health reform | Focused solely on service delivery or outreach components, i.e., not focused on social participation for broader health reform /priority-setting |
| **Outcome(s)*** | Provides information on outcome(s) of processes for social participation for health reform—including organisational process outcomes, community process outcomes, community outcomes, health outcomes, stakeholder perspectives on participatory processes— degree of inclusiveness, and/or how inclusiveness influenced the outcomes of social participation for health reform | Focused only on population level outcomes or impacts that are not directly or indirectly linked to processes for social participation in health reform (e.g., greater enrollment of a population in health reform that was not involved in social participation processes) |
| **Study design(s)** | Peer-reviewed or grey empirical sources | Conference abstracts, theses/dissertations, and non-empirical sources (e.g., opinion papers, guidelines, policies, editorials) |
| **Type of data** | Qualitative and/or quantitative | N/A |
| **Language of publication** | English or Spanish | Not English or Spanish |
| **Year of publication** | 2000-2023 | Before 2000 |

Following a team-wide pilot screen that achieved 75% agreement through double screening, titles and abstracts were screened by a single reviewer. In contrast, Spanish-language articles underwent double screening, as the pilot test did not reach the 75% agreement threshold. At the full-text screening stage, both English and Spanish articles underwent double screening given the inability to achieve 75% agreement in a pilot stage. Screening conflicts were resolved by a third independent reviewer.

**3. Appraisal of studies and extraction of data**

Full-text appraisal was conducted to assess the quality and relevance of articles for inclusion (see Table 2). Quality appraisal was adapted from Hebbar et al. (2022) and applied using a scoring range of 0–5, where criteria met were scored as 1 and criteria unmet or insufficiently detailed were scored as 0 (Hebbar et al. 2022). For the relevance appraisal, the research team developed criteria based on the research question, following the same 0–5 scoring approach used for the quality appraisal. Articles scoring 3 or higher for both rigor and relevance were included for full-text analysis and data extraction.

**Table 2. Quality appraisal criteria**

| **Methodological reporting and rigour** | **Score (0 or 1)** |
| --- | --- |
| **Objectives/research questions are clearly defined** |  |
| **Sampling explained** |  |
| **Data gathering process explained** |  |
| **Data analysis explained** |  |
| **Coherence between objective, methods, and findings** |  |
|  | **Total: 5** |
| **Relevance**  The article explains: | **Score (0 or 1)** |
| **Strategies, activities, and/or resources used in SPH**  *where* ***SPH*** *can involve ‘claimed’ spaces leading to health reform or the creation of ‘invited’ spaces as part of a health reform, and* ***health reform*** *is any change to the six WHO health system building blocks and/or a policy change addressing the social determinants of health* |  |
| **Actors (e.g., individuals, communities, organizations) involved in SPH** |  |
| **Strategies, activities, and/or resources to promote SPH’s inclusiveness**  *with* ***inclusiveness*** *encompassing numeric diversity, the extent of meaningful participation by people experiencing marginalization, and/or experiences of inclusion or exclusion* |  |
| **Outcomes of efforts to promote inclusiveness in SPH (i.e., whether inclusiveness was achieved, to what extent, etc.)** |  |
| **Outcomes associated with inclusiveness of SPH (i.e., costs, organizational processes, community processes, community outcomes, health outcomes, perspectives, and/or empowerment, as operationalized by (Haldane et al. 2019).** |  |
|  | **Total: 5** |

Blinded double appraisal was performed in Covidence for both English and Spanish articles, with conflicts resolved by a third reviewer.

Data extraction was managed in Covidence and included variables such as title, authors, publication year, country of focus, study aims (explicit and implicit), article type, types of knowledge generation, description and timeline of health reforms, focus and strategies for social participation, and resources for enhancing inclusiveness in participation.

Single data extraction was employed for English articles due to the high volume of retrieved studies, while double data extraction was employed for Spanish articles. A third team member conducted side-by-side comparisons of both reviewers’ extractions for Spanish articles, reconciling differences to produce the final dataset.

4. **Synthesis of evidence and drawing of conclusions**

Analysis is taking place in a two-stage process. In the first stage, hybrid content analysis (in preparation for subsequent analyses) was conducted. In the second stage, context-mechanism-outcome configurations are being generated.

**Search strategy**

# Table 3. Subject headings and key words

| **PubMed** | **Embase** | **CINAHL** | **Other Key Words (Synonyms)** | **Spanish Equivalents** |
| --- | --- | --- | --- | --- |
| **Social participation string** | | | | |
| Social participation | Social participation/ | Social participation |  |  |
| Stakeholder participation | Stakeholder engagement/ | Stakeholder participation | Stakeholder involvement |  |
| Community participation | Community participation/ | (used as keyword) | Community engagement |  |
| Decision making, shared | Shared decision making | Shared decision making |  |  |
| Citizen science | Citizen science | Citizen science |  |  |
| Social responsibility | Social responsibility | Social responsibility |  |  |
| Community networks | Community care (covers Mesh term in addition to other terms) | Community networks |  |  |
| Cooperative behaviour | Cooperation | Cooperative behaviour |  |  |
| Consumer advocacy | Consumer advocacy | Consumer advocacy |  |  |
| Community-industrial relations | Community Outreach | Community Outreach |  | |
| **Health reform string** | | | | |
| Universal health care | Universal health care | Universal health care |  |  |
| Universal health insurance | Universal health insurance | Insurance, health, reimbursement    Insurance, health |  |  |
| Health care reform | Health care policy (covers Mesh term in addition to other terms) | Health care reform |  |  |
| Health priorities | Health care planning (covers Mesh term in addition to other terms) | Health priorities |  |  |
| Health policy | Health care policy (covers Mesh term in addition to other terms; also a duplicate with “health care reform”) | Health policy |  |  |
| National health programs | Public health (covers Mesh term in addition to other terms) | National health programs |  |  |
| Policy making | Management (covers Mesh term in addition to other terms) | Policy making |  |  |
| Decision making, organizational | Organizational decision making | Decision making, organizational |  |  |
| Public policy | Public policy | Public policy |  |  |
| Health care rationing | Health care organization (covers Mesh term in addition to other terms) | Health resource allocation |  |  |
| Health planning | Health care planning (also a duplicate with “health priorities”) | Health and welfare planning |  |  |
| **Health equity string** | | | | |
| Right to health | Right to health | Right to health |  |  |
| Social inclusion | Social inclusion | Social inclusion |  |  |
| Diversity, equity, inclusion    Cultural diversity    Ethnic and racial minorities/    Gender equity/ | Cultural diversity    Racial diversity/    Ethnic group/    Gender diversity/ | Cultural diversity    Racial equality/    Ethnic group/    Gender equality/ | equit* adj1 divers*    divers* adj1 inclus*    equit* adj1 inclus*    "EDI" or "ED&I"    "DEI" or "DE&I" |  |
| Intersex persons/ | Add as key word | Intersex persons/ |  | |
| Transgender persons/ | Transgender persons/ | Transgender persons/ |  | |
| Health inequities    Healthcare disparities/ | Health disparity/ (covers Mesh term in addition to other terms)    Health inequity/ | Health inequities |  |  |
| Health disparate, minority and vulnerable populations + all subcategory terms covering racial and ethnic groups, sexual and gender minorities and vulnerable populations (see comment on the side) | Vulnerable population/    “sexual and gender minority”/ | Special population/    Sexual and gender minorities/ |  |  |
| Health equity | Health equity | Health equity (keyword) |  |  |
| Minority groups | Minority group | Minority groups |  |  |
| Social marginalization | Social exclusion | "social* marginaliz*" (added as a key term) |  |  |
| Social vulnerability | Social vulnerability | Vulnerability    "social* vulnerab*" (added as a key term) |  |  |
| Minority health | Minority health | Minority health (keyword) |  |  |
| Social determinants of health | Social determinants of health | Social determinants of health |  |  |
| Low socioeconomic status | Lowest income group/    Working poor/ | - | Low* socioeconomic    Low* socio economic |  |
| Healthcare disparities | Health disparity (duplicate above with health inequities) | Healthcare disparities |  |  |
| Developing countries | Developing country | Developing countries |  |  |
|  | Middle income country/ | Low and middle income countries/ | LMIC*    Low income countr*    Middle income countr*    Low and middle income countr* |  |
| Poverty | Poverty    Extreme poverty    Poverty level | Poverty |  |  |
| Social deprivation | Social isolation (covers Mesh term in addition to other terms) | Social deprivation |  |  |
| Cultural deprivation | Cultural deprivation | Cultural deprivation |  |  |

##

## **Table 4. Medline (PubMed)**

| **Date** | **#** | **Search Terms** | **Hits** |
| --- | --- | --- | --- |
| 22-05-2023 | 1 | social participation.mp. or Social Participation/ or stakeholder participation.mp. or Stakeholder Participation/ or stakeholder engagement.mp. or stakeholder involvement.mp. or community participation.mp. or Community Participation/ or community engagement.mp. or shared decision making.mp. or Decision Making, Shared/ or citizen science.mp. or Citizen Science/ or social responsibility.mp. or Social Responsibility/ or community network*.mp. or Community Networks/ or cooperative behavio?r.mp. or Cooperative Behavior/ or cooperative.mp. or Consumer Advocacy/ or consumer advoca*.mp. | 191601 |
|  | 2 | (universal healthcare or universal health care).mp. or Universal Health Care/ or universal health insurance.mp. or Universal Health Insurance/ or (healthcare reform or health care reform).mp. or Health Care Reform/ Or health priorities.mp. or Health Priorities/ or health planning.mp. or Health Planning/ or (healthcare planning or health care planning).mp. or health policy.mp. or Health Policy/ or (healthcare policy or health care policy).mp. or National Health Programs/ or national health program*.mp. or public health.mp. or Public Health/ or (policy making or policymaking).mp. or organizational decision making.mp. or Decision Making, Organizational/ or public policy.mp. or Public Policy/ or Health Care Rationing/ or (healthcare rationing or health care rationing).mp. or (health care organization or healthcare organization).mp. or health resource allocation.mp. | 663316 |
|  | 3 | 1 AND 2 | 24797 |
|  | 4 | right to health.mp. or Right to Health/ or social inclusion.mp. or Social Inclusion/ or cultural diversity/ or diversity, equity, inclusion/ or (ethnic minorit* or racial minorit*).mp. or "Ethnic and Racial Minorities"/ or gender equity.mp. or Gender Equity/ or (equit* adj1 divers*).mp. or (divers* adj1 inclus*).mp. or (equit* adj1 inclus*).mp. or ("EDI" or "ED&I").mp. or ("DEI" or "DE&I").mp. or Health Inequities/ or health* inequit*.mp. Or Healthcare Disparities/ or health* disparit*.mp. or health equity.mp. or Health Equity/ or Minority Groups/ or minority group*.mp. or Social Marginalization/ or social* marginaliz*.mp. or social exclusion.mp. or Social Vulnerability/ or social* vulnerab*.mp. or Minority Groups/ or minority health*.mp. or social determinant* of health.mp. or "Social Determinants of Health"/ or (Low* socioeconomic or Low* socio economic).mp. or low socioeconomic status/ or Developing Countries/ or developing countr*.mp. or (middle income countr* or low income countr* or LMIC* or "low and middle income countr*").mp. or poverty/ or child poverty/ or (poverty or child poverty).mp. or social deprivation.mp. or Social Deprivation/ or cultural deprivation.mp. or Cultural Deprivation/ or "health disparate, minority and vulnerable populations"/ or "black or african american"/ or amish/ or arabs/ or "asian american native hawaiian and pacific islander"/ or "hispanic or latino"/ or indigenous peoples/ or jews/ or roma/ or "sexual and gender minorities"/ or vulnerable populations/ or vulnerable population*.mp. or (sexual minorit* or gender minorit).mp. | 495986 |
|  | 5 | 3 AND 4 | 4197 |
|  | 6 | limit to yr="2000 - 2023" | 3040 |

## **Table 5. Embase**

| **Date** | **#** | **Search Terms** | **Hits** |
| --- | --- | --- | --- |
| 22-05-2023 | 1 | social participation.mp. OR social participation/ OR stakeholder engagement.mp. OR stakeholder engagement/ OR stakeholder participation.mp. OR stakeholder involvement.mp. OR community participation/ OR community engagement.mp. OR shared decision making.mp. OR shared decision making/ OR citizen science.mp. OR citizen science/ OR social responsibility.mp. OR social responsibility/ OR community care/ OR community network*.mp. OR cooperation/ OR cooperative behavio?r.mp. OR consumer advocacy/ OR consumer advoca*.mp. | 170,097 |
|  | 2 | (universal healthcare OR universal health care).mp. OR universal health care/ OR (health policy OR healthcare policy OR health care policy).mp. OR health care policy/ OR (healthcare reform OR health care reform).mp. OR (healthcare planning OR health care planning).mp. OR health care planning/ OR health priorities.mp. OR national health program*.mp. OR public health/ OR management/ OR (policymaking OR policy making).mp. OR organizational decision making.mp. OR organizational decision making/ OR public policy.mp. OR public policy/ OR health care organization.mp. OR health care organization/ OR (healthcare rationing OR health care rationing).mp. OR health resource allocation.mp. OR health planning.mp. OR (health and welfare planning).mp. | 722,891 |
|  | 3 | 1 AND 2 | 28,175 |
|  | 4 | right to health.mp. or right to health/ or social inclusion.mp. or social inclusion/ or cultural diversity.mp. or cultural diversity/ or racial diversity.mp. or racial diversity/ or ethnic group.mp. or ethnic group/ or gender diversity.mp. or gender diversity/ or (equit* adj1 divers*).mp. or (divers* adj1 inclus*).mp. or (equit* adj1 inclus*).mp. or ("EDI" or "ED&I").mp. or ("DEI" or "DE&I").mp. or health disparity.mp. or health disparity/ or health inequity/ or health inequit*.mp. or vulnerable population/ or vulnerable population*.mp. or "sexual and gender minorities".mp. or "sexual and gender minority"/ or health equity.mp. or health equity/ or minority group.mp. or minority group/ or social exclusion.mp. or social exclusion/ or "social* marginaliz*".mp. or social vulnerability/ or social* vulnerab*.mp. or minority health.mp. or minority health/ or social determinant* of health.mp. or "social determinants of health"/ or lowest income group.mp. or lowest income group/ or working poor.mp. or working poor/ or (Low* socioeconomic or Low* socio economic).mp. or developing country/ or developing countr*.mp. or middle income country/ or middle income countr*.mp. or low income countr*.mp. or (Low and middle income countr*).mp. or LMIC*.mp. or poverty.mp. or poverty/ or extreme poverty.mp. or extreme poverty/ or child poverty.mp. or child poverty/ or poverty level.mp. or poverty level/ or social isolation.mp. or social isolation/ or social deprivation.mp. or cultural deprivation.mp. or cultural deprivation/ | 578780 |
|  | 5 | 3 AND 4 | 4398 |
|  | 6 | limit 5 to yr="2000 - 2023" | 4,067 |

## **Table 6. CINAHL**

| **Date** | **#** | **Search Terms** | **Hits** |
| --- | --- | --- | --- |
| 22-05-2023 | 1 | (MH "Social Participation") OR "social participation" OR (MH "Stakeholder Participation") OR "stakeholder participation" OR "stakeholder engagement" OR "stakeholder involvement" OR "community participation" OR "community engagement" OR (MH "Decision Making, Shared") OR "shared decision making" OR (MH "Citizen Science") OR "citizen science" OR (MH "Social Responsibility") OR "social responsibility" OR (MH "Community Networks") OR "community network*" OR (MH "Cooperative Behavior") OR "cooperative behaviour or cooperative behavior" OR (MH "Consumer Advocacy") OR "consumer advoca*" | 50,184 |
|  | 2 | (MH "Universal Health Care") OR "universal healthcare or universal health care" OR (MH "Insurance, Health, Reimbursement") OR (MH "Insurance, Health") OR "universal health insurance" OR (MH "Health Care Reform") OR "healthcare reform or health care reform" OR "healthcare policy or health care policy or health policy" OR (MH "Health Priorities") OR "health priorities" OR (MH "National Health Programs") OR "national health program*" OR (MH "Public Health") OR "public health" OR (MH "Policy Making") OR "policymaking or policy making" OR (MH "Decision Making, Organizational") OR "organizational decision making" OR (MH "Public Policy") OR "public policy" OR (MH "Health Resource Allocation") OR "health resource allocation" OR "healthcare rationing or health care rationing" OR "healthcare organization or health care organization" OR (MH "Health and Welfare Planning") OR "health and welfare planning" OR "health planning or healthcare planning or health care planning" | 885,856 |
|  | 3 | 1 AND 2 | 14,262 |
|  | 4 | (MH "Right to Health") OR "right to health" OR (MH "Social Inclusion") OR "social inclusion" OR (MH "Cultural Diversity") OR "cultural diversity" OR (MH "Racial Equality") OR "racial equality" OR "ethnic group" OR (MH "Gender Equality") OR "gender equality" OR "equit* N1 divers*" OR "divers* N1 inclus*" OR "equit* N1 inclus*" OR ""EDI" or "ED&I"" OR ""DEI" or "DE&I"" OR (MH "Health Inequities") OR "health inequit*" OR "health disparit*" OR "special population*" OR (MH "Sexual and Gender Minorities") OR "sexual minorit* or gender minorit*" OR (MH "Minority Groups") OR "minority group*" OR "health equit*" OR ""social* marginaliz*"" OR "social exclusion" OR ""social* vulnerab*"" OR (MH "Vulnerability") OR "vulnerability" OR "minority health" OR (MH "Social Determinants of Health") OR "social determinant* of health" OR "Low* socioeconomic or Low* socio economic or working poor or low* income" OR (MH "Developing Countries") OR "developing countr*" OR (MH "Low and Middle Income Countries") OR "low income countr* or middle income countr* or low and middle income countr* or LMIC*" OR (MH "Poverty") OR "poverty" OR (MH "Social Deprivation") OR "social deprivation" OR (MH "Cultural Deprivation") OR "cultural deprivation"  **Note:** The subject heading “social inclusion” is not a perfect match with the MeSH term. In MeSH, “social inclusion” refers to “the process of improving the basis on which individuals and groups take part in society by improving the ability, opportunity, and dignity of those disadvantaged” but in CINHAL the scope note is as follows: “The extent to which people experience a sense of belonging within their social environment.” One is centered more on “process” while the other more on “experience” of individuals. However, this term was still included in CINAHL as the two subject headings were still similar, and adding this term did not explode the results too much. | 188,791 |
|  | 5 | 3 and 4 | 2323 |
|  | 6 | Restrict to January 1, 2000 – December 31, 2023 | 2,256 |

# Table 7. Web of Science – Social Sciences Citation Index (SSCI)

| **Date** | **#** | **Search Terms** | **Hits** |
| --- | --- | --- | --- |
| 22-05-2023 | 1 | (stakeholder* OR participant* or community) NEAR/1 (involve* or engag* or empower* or participation) or “shared decision making” or “shared decision-making” or “citizen science” or “social responsibility” or “community network*” or “cooperative behaviour” or “cooperative behavior” or “cooperation” or “collaboration” or “consumer advoca*” | 202,522 |
|  | 2 | “universal health care” or “universal healthcare” or “universal health insurance” or “healthcare reform” or “health care reform” or “healthcare policy” or “health care policy” or “health policy” or “health care planning” or “healthcare planning” or “national health program*” or “public health” or “policymaking” or “policy making” or “organizational decision making” or “organizational decision-making” or “public policy” or “healthcare rationing” or “health care rationing” or “health resource allocation” or “health* planning” | 209, 768 |
|  | 3 | 1 AND 2 | 10,154 |
|  | 4 | “right to health” or “social inclusion” or “cultural diversity” or “racial diversity” or “ethnic diversity” or “gender equity” or equit* NEAR/1 divers* OR divers* NEAR/1 inclus*OR equit* NEAR/1 inclus* OR "EDI" or "ED&I" OR "DEI" or "DE&I" OR “health* inquit*” or “health* disparit*” or “health equity” or “vulnerable population*” or “sexual minorit*” or “gender minorit*” or “minority group*” or “social* marginaliz*” or "social* vulnerab*" or “minority health” or “social determinant* of health” or “Low* socioeconomic” or “Low* socio economic” or “developing countr*” or “LMIC*” or “Low income countr*” or “Middle income countr*” or “Low and middle income countr*” or “poverty” or “social deprivation” or “cultural deprivation” | 225,438 |
|  | 5 | 3 AND 4 | 1782 |
|  | 6 | Time bounds for publication year: 2000-2023 | 1757 |

#

# Table 8. ProQuest Theses and Dissertations A&I

| **Date** | **#** | **Search Terms** | **Hits** |
| --- | --- | --- | --- |
| 22-05-2023 | 1 | (stakeholder* OR participant* OR community) NEAR/1 (involve* OR engag* OR empower* OR participation) OR "shared decision making" OR "shared decision-making" OR "citizen science" OR "community network*" OR "consumer advoca*" OR "participat*" | 204,587 |
|  | 2 | "universal health care" OR "universal healthcare" OR "universal health insurance" OR "healthcare reform" OR "health care reform" OR "health* policy" OR "health care planning" OR "healthcare planning" OR "national health program*" OR "policymaking" OR "policy making" OR "healthcare rationing" OR "health care rationing" OR "health resource allocation" OR "health* planning" OR "public health" | 35,875 |
|  | 3 | 1 AND 2 | 4632 |
|  | 4 | "right to health" OR "social inclusion" OR "cultural diversity" OR "racial diversity" OR "ethnic diversity" OR "gender equity" OR equit* NEAR/1 divers* OR divers* NEAR/1 inclus*OR equit* NEAR/1 inclus* OR "EDI" OR "ED&I" OR "DEI" OR "DE&I" OR "health* inquit*" OR "health* disparit*" OR "health equity" OR "vulnerable population*" OR "sexual minorit*" OR "gender minorit*" OR "minority group*" OR "social* marginaliz*" OR "social* vulnerab*" OR "minority health" OR "social determinant* of health" OR "Low* socioeconomic" OR "Low* socio economic" OR "developing countr*" OR "LMIC*" OR "low income countr*" OR "middle income countr*" OR "Low and middle income countr*" OR "poverty" OR "social deprivation" OR "cultural deprivation" | 59,684 |
|  | 5 | 3 AND 4 | 706 |
|  | 6 | Time bounds for publication year: 2000-2023 | 641 |

# Table 9. PDQ

| **Date** | **#** | **Search Terms (Combined with ‘OR’)** | **Hits** |
| --- | --- | --- | --- |
| 13-05-2023 | 1 | Engag*  Participat*  Stakeholder*  Decision*making  Citizen science  Cooperat*  Collaborat*  Advoca*  community network*  social responsibilit*    (title:(engag*) OR abstract:(engag*)) OR (title:(participat*) OR abstract:(participat*)) OR (title:(stakeholder*) OR abstract:(stakeholder*)) OR (title:(decision*making) OR abstract:(decision*making)) OR (title:(citizen science) OR abstract:(citizen science)) OR (title:(cooperat*) OR abstract:(cooperat*)) OR (title:(collaberat*) OR abstract:(collaberat*)) OR (title:(advoca*) OR abstract:(advoca*)) OR (title:(community network*) OR abstract:(community network*)) OR (title:(social responsibilit*) OR abstract:(social responsibilit*)) | 19,509 |
|  | 2 | Universal health care  Universal healthcare  Universal health coverage  Universal health insurance  Policy  Planning  Management  Resource allocation  Health plan*  Health program*  Welfare  Reform*  Medicare  Health* rationing  Health* financing    (title:(universal health coverage) OR abstract:(universal health coverage)) OR (title:(universal health insurance) OR abstract:(universal health insurance)) OR (title:(universal health care) OR abstract:(universal health care)) OR (title:(universal healthcare) OR abstract:(universal healthcare)) OR (title:(policy) OR abstract:(policy)) OR (title:(planning) OR abstract:(planning)) OR (title:(management) OR abstract:(management)) OR (title:(resource allocation) OR abstract:(resource allocation)) OR (title:(health plan*) OR abstract:(health plan*)) OR (title:(health program*) OR abstract:(health program*)) OR (title:(welfare) OR abstract:(welfare)) OR (title:(reform*) OR abstract:(reform*)) OR (title:(medicare) OR abstract:(medicare)) OR (title:(health* rationing) OR abstract:(health* rationing)) OR (title:(health* financing) OR abstract:(health* financing)) | 35,830 |
|  | 3 | 1 AND 2 | 8,799 |
|  | 4 | Right to health  Human right*  Equity  equality  health disparit*  inequit*  inclusion  social determinant* of health  developing countr*  LMIC*  low and middle income countr*  poverty  socio*economic status  social status  minorit*  divers*  Vulnerab*  marginaliz*  ED*I  DE*I    (title:(right to health) OR abstract:(right to health)) OR (title:(human right*) OR abstract:(human right*)) OR (title:(equity) OR abstract:(equity)) OR (title:(health disparit*) OR abstract:(health disparit*)) OR (title:(inclusion) OR abstract:(inclusion)) OR (title:(social determinant* of health) OR abstract:(social determinant* of health)) OR (title:(cultural deprivation) OR abstract:(cultural deprivation)) OR (title:(developing countr*) OR abstract:(developing countr*)) OR (title:(poverty) OR abstract:(poverty)) OR (title:(socio*economic status) OR abstract:(socio*economic status)) OR (title:(minorit*) OR abstract:(minorit*)) OR (title:(divers*) OR abstract:(divers*)) OR (title:(Vulnerab*) OR abstract:(Vulnerab*)) OR (title:(Neurodivers*) OR abstract:(Neurodivers*)) OR (title:(equality) OR abstract:(equality)) OR (title:(social status) OR abstract:(social status)) OR (title:(LMIC*) OR abstract:(LMIC*)) OR (title:(low AND middle income countr*) OR abstract:(low AND middle income countr*)) OR (title:(marginaliz*) OR abstract:(marginaliz*)) OR (title:(inequit*) OR abstract:(inequit*)) OR (title:(ED*I) OR abstract:(ED*I)) OR (title:(DE*I) OR abstract:(DE*I)) | 16,448 |
|  | 5 | 3 AND 4 | 2,070 |
|  | 6 | Restricted to year 2000-2023    (title:((title:(engag*) OR abstract:(engag*)) OR (title:(participat*) OR abstract:(participat*)) OR (title:(stakeholder*) OR abstract:(stakeholder*)) OR (title:(decision*making) OR abstract:(decision*making)) OR (title:(citizen science) OR abstract:(citizen science)) OR (title:(cooperat*) OR abstract:(cooperat*)) OR (title:(collaberat*) OR abstract:(collaberat*)) OR (title:(advoca*) OR abstract:(advoca*)) OR (title:(community network*) OR abstract:(community network*)) OR (title:(social responsibilit*) OR abstract:(social responsibilit*))) OR abstract:((title:(engag*) OR abstract:(engag*)) OR (title:(participat*) OR abstract:(participat*)) OR (title:(stakeholder*) OR abstract:(stakeholder*)) OR (title:(decision*making) OR abstract:(decision*making)) OR (title:(citizen science) OR abstract:(citizen science)) OR (title:(cooperat*) OR abstract:(cooperat*)) OR (title:(collaberat*) OR abstract:(collaberat*)) OR (title:(advoca*) OR abstract:(advoca*)) OR (title:(community network*) OR abstract:(community network*)) OR (title:(social responsibilit*) OR abstract:(social responsibilit*)))) AND (title:((title:(universal health coverage) OR abstract:(universal health coverage)) OR (title:(universal health insurance) OR abstract:(universal health insurance)) OR (title:(universal health care) OR abstract:(universal health care)) OR (title:(universal healthcare) OR abstract:(universal healthcare)) OR (title:(policy) OR abstract:(policy)) OR (title:(planning) OR abstract:(planning)) OR (title:(management) OR abstract:(management)) OR (title:(resource allocation) OR abstract:(resource allocation)) OR (title:(health plan*) OR abstract:(health plan*)) OR (title:(health program*) OR abstract:(health program*)) OR (title:(welfare) OR abstract:(welfare)) OR (title:(reform*) OR abstract:(reform*)) OR (title:(medicare) OR abstract:(medicare)) OR (title:(health* rationing) OR abstract:(health* rationing)) OR (title:(health* financing) OR abstract:(health* financing))) OR abstract:((title:(universal health coverage) OR abstract:(universal health coverage)) OR (title:(universal health insurance) OR abstract:(universal health insurance)) OR (title:(universal health care) OR abstract:(universal health care)) OR (title:(universal healthcare) OR abstract:(universal healthcare)) OR (title:(policy) OR abstract:(policy)) OR (title:(planning) OR abstract:(planning)) OR (title:(management) OR abstract:(management)) OR (title:(resource allocation) OR abstract:(resource allocation)) OR (title:(health plan*) OR abstract:(health plan*)) OR (title:(health program*) OR abstract:(health program*)) OR (title:(welfare) OR abstract:(welfare)) OR (title:(reform*) OR abstract:(reform*)) OR (title:(medicare) OR abstract:(medicare)) OR (title:(health* rationing) OR abstract:(health* rationing)) OR (title:(health* financing) OR abstract:(health* financing)))) AND (title:((title:(right to health) OR abstract:(right to health)) OR (title:(human right*) OR abstract:(human right*)) OR (title:(equity) OR abstract:(equity)) OR (title:(health disparit*) OR abstract:(health disparit*)) OR (title:(inclusion) OR abstract:(inclusion)) OR (title:(social determinant* of health) OR abstract:(social determinant* of health)) OR (title:(cultural deprivation) OR abstract:(cultural deprivation)) OR (title:(developing countr*) OR abstract:(developing countr*)) OR (title:(poverty) OR abstract:(poverty)) OR (title:(socio*economic status) OR abstract:(socio*economic status)) OR (title:(minorit*) OR abstract:(minorit*)) OR (title:(divers*) OR abstract:(divers*)) OR (title:(Vulnerab*) OR abstract:(Vulnerab*)) OR (title:(Neurodivers*) OR abstract:(Neurodivers*)) OR (title:(equality) OR abstract:(equality)) OR (title:(social status) OR abstract:(social status)) OR (title:(LMIC*) OR abstract:(LMIC*)) OR (title:(low AND middle income countr*) OR abstract:(low AND middle income countr*)) OR (title:(marginaliz*) OR abstract:(marginaliz*)) OR (title:(inequit*) OR abstract:(inequit*)) OR (title:(ED*I) OR abstract:(ED*I)) OR (title:(DE*I) OR abstract:(DE*I))) OR abstract:((title:(right to health) OR abstract:(right to health)) OR (title:(human right*) OR abstract:(human right*)) OR (title:(equity) OR abstract:(equity)) OR (title:(health disparit*) OR abstract:(health disparit*)) OR (title:(inclusion) OR abstract:(inclusion)) OR (title:(social determinant* of health) OR abstract:(social determinant* of health)) OR (title:(cultural deprivation) OR abstract:(cultural deprivation)) OR (title:(developing countr*) OR abstract:(developing countr*)) OR (title:(poverty) OR abstract:(poverty)) OR (title:(socio*economic status) OR abstract:(socio*economic status)) OR (title:(minorit*) OR abstract:(minorit*)) OR (title:(divers*) OR abstract:(divers*)) OR (title:(Vulnerab*) OR abstract:(Vulnerab*)) OR (title:(Neurodivers*) OR abstract:(Neurodivers*)) OR (title:(equality) OR abstract:(equality)) OR (title:(social status) OR abstract:(social status)) OR (title:(LMIC*) OR abstract:(LMIC*)) OR (title:(low AND middle income countr*) OR abstract:(low AND middle income countr*)) OR (title:(marginaliz*) OR abstract:(marginaliz*)) OR (title:(inequit*) OR abstract:(inequit*)) OR (title:(ED*I) OR abstract:(ED*I)) OR (title:(DE*I) OR abstract:(DE*I)))) | 1982 |

# Characteristics of included studies

**Figure 1: PRISMA Chart**

**
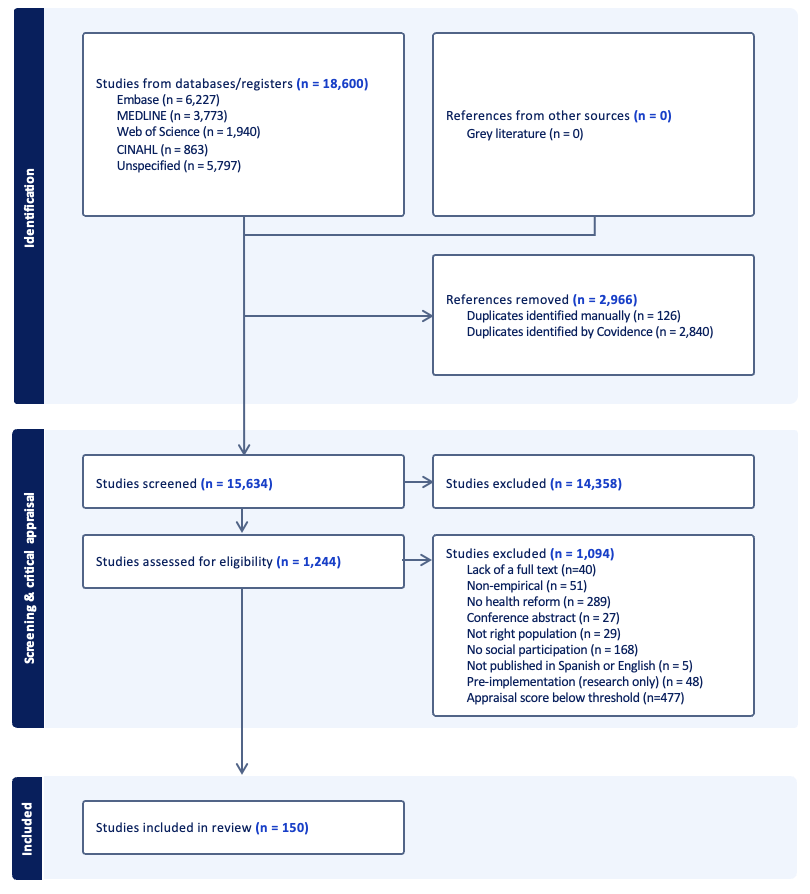
**

**Figure 2: Frequency of countries of focus in SPH included studies**


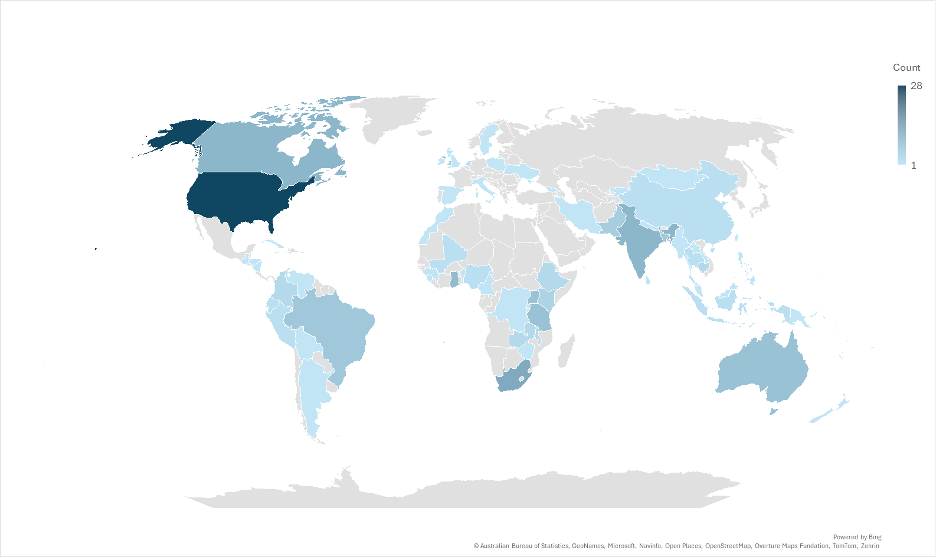


**Table 10. Summary of included studies**

| **Title** | **Author-date reference** | **Country or countries of focus** | **Study aim(s)** | **Type(s) of knowledge generation** |
| --- | --- | --- | --- | --- |
| Beyond positive a priori bias: reframing community engagement in LMICs | Abimbola 2020 | Nigeria | To draw upon theory and empirical evidence to demonstrate that understanding of why, when and by which mechanisms community engagement emerges is critical to bolster community engagement in primary health care. | Analysis of existing data (secondary analysis); Evidence synthesis (i.e. review) |
| El caso de la implementación de la estrategia de Atención Primaria Integral en Salud (APIS) en la localidad de Suba, Bogotá – Colombia | Acosta Ramirez 2008 | Colombia | To provide a situational analysis of the experience of implementing a comprehensive primary care strategy within Suba, Colombia. | Primary data collection; Analysis of existing data (secondary analysis) |
| Coordination of the health policy dialogue process in Guinea: pre- and post-Ebola | Ade 2016 | Guinea | Investigate the process of the policy dialogue used to revise Guinea's national health policy and develop Guinea's national health development plan for 2015-2024, including whether/how the Ebola epidemic shaped the policy dialogue's coordination | Primary data collection |
| Knowledge of health rights and community participation in primary care | Agegnehu 2022 | Ethiopia | To evaluate community health leaders' knowledge related to health rights and community participation in an Ethiopia town. | Primary data collection |
| Los Inmigrantes en El Sistema Sanitario Español: Aspectos Participativos Y de Atencion a la Salud | AguilarGil 2019 | Spain | To identify participatory processes within the Spanish healthcare system involving immigrants, assess their level of involvement, observe the differences between the various processes, and propose improvements. | Primary data collection |
| Stakeholder perceptions on scaling-up community-led interventions for prevention and control of non-communicable diseases in Bangladesh: a qualitative study | Akter 2023 | Bangladesh | To determine the mechanism(s) by which a successful type 2 diabetes community-led intervention based in rural Bangladesh might be scaled up. | Primary data collection |
| Challenges to achieving universal health coverage through community-based health planning and services delivery approach: a qualitative study in Ghana | Assan 2019 | Ghana | To discern any challenges to universal health coverage achievement via Ghana's community-based health planning and service initiative. | Primary data collection |
| Willing but unable? Extending theory to investigate community capacity to participate in Ghana's Community-based Health Planning and service implementation | Atinga 2019 | Ghana | To assess community capacity for participating in the Community-Based Health Planning and Service (CHPS) within Ghana. | Primary data collection |
| The architecture and effect of participation: A systematic review of community participation for communicable disease control and elimination. Implications for malaria elimination | Atkinson 2011 | No geographical restrictions for study selection; included studies covered Argentina, Bolivia, Brazil, Cambodia, Cameroon, China, Colombia, Cuba, Cyprus, Ethiopia, Guatemala, Honduras, India, Indonesia, Kenya, Laos, Malaysia, Mali, Morocco, Nicaragua, Nigeria, Pakistan, Papua New Guinea, Peru, South Africa, Sri Lanka, Taiwan, Tanzania, Thailand, Uganda, USA, Vanuatu, Venezuela, Zaire, Zimbabwe | To synthesize and draw guidance from literature with the aim of understanding community participation for controlling and eliminating communicable diseases | Evidence synthesis (i.e. review) |
| Community Health Worker Activities in Public Health Programs to Prevent Violence: Coding Roles and Scope | Barbero 2022 | USA | To analyze recent public health CHW initiatives in the area of violence prevention, in order to inform the development of programs and research in future. | Analysis of existing data (secondary analysis) |
| Community participation in primary health care projects of the Muldersdrift Health and Development Programme | Barker 2007 | South Africa | To examine the level of community participation within the primary health care projects of the Muldersdrift Health and Development Programme. | Primary data collection |
| Social participation in health in Brazil and England: inclusion, representation and authority. | Barnes 2009 | Brazil, UK (England) | To describe and examine public participation in health in Brazil and England in order to highlight different motivators and tensions within an acceptance of participation as official policy |  |
| Challenges to the utilization of Community-based Health Planning and Services: the views of stakeholders in Yendi Municipality, Ghana | Bassoumah 2021 | Ghana | To explore the implementation and utilization challenges of the Community-based Health Planning and Services (CHPS) programme in the Northern Region of Ghana. | Primary data collection |
| Does the provision of community health services offset the effects of poverty and low maternal educational attainment on childhood mortality? An analysis of the equity effect of the Navrongo experiment in Northern Ghana | Bawah 2019 | Ghana | To evaluate the long-term impact of community health worker exposure among children. | Analysis of existing data (secondary analysis) |
| The child survival impact of the Ghana Essential Health Interventions Program: A health systems strengthening plausibility trial in Northern Ghana | Bawah 2019 | Ghana | To examine the impacts of the Ghana Essential Health Interventions Program on child survival, alongside the factors associated with such impacts. | Primary data collection |
| The Rohingya Little Local: exploring innovative models of refugee engagement in Sydney, Australia | Bestman 2020 | Australia | To investigate the potential of the community-led funding model as well as the impact of the model on health services and community. | Primary data collection; Analysis of existing data (secondary analysis) |
| Atencion primaria social en Colombia: una mirada desde una experiencia exitosa | Betancurth-Loaiza 2022 | Colombia | To analyze the experience of Social Primary Healthcare in the Department of Caldas as part of a systematization process. | Analysis of existing data (secondary analysis) |
| Unlocking community capability through promotion of self-help for health: experience from Chakaria, Bangladesh | Bhuiya 2016 | Bangladesh | To outline the implementation, impact and learnings related to a self-help health project in Bangladesh. | Primary data collection |
| Improving policy and practice to promote equity and social justice - a qualitative comparative analysis building on key learnings from a twinning exchange between England and the US | Blanchard 2013 | UK (England), USA | To distill lessons surrounding community interventions within England and the USA, drawing upon a learning exchange that took place between Communities for Health in England and the Racial and Ethnic Approaches to Community Health across the USA. | Primary data collection |
| [Building the social health movement in Colombia, 1998-2020]. | Borrero-Ramirrez 2021 | Colombia | Analyzes the relationship between state reforms, health policy changes, and the stages of the social movement for health in Colombia, seeking to establish a dialogue between structural transformations, the configuration of collective actors, and contested struggles. | Primary data collection; Analysis of existing data (secondary analysis) |
| A Comprehensive Review of Optimal Approaches to Co-Design in Health with First Nations Australians | Butler 2022 | Australia | This project aimed to identify the current evidence around optimal approaches to co-design in health with First Nations Australians. | Evidence synthesis (i.e. review) |
| Diagnóstico de situación y evaluación de un programa de educación comunitaria para la prevención del Síndrome Urémico Hemolítico | Caletti 2009 | Argentina | The main objectives were: (1) to carry out a baseline assessment of the local situation prior to program implementation through operational research, and (2) to evaluate the outcomes of the intervention through a process evaluation. | Analysis of existing data (secondary analysis) |
| Abordajes comunitarios de los consumos problemáticos de drogas | Camarrotti 2016 | Argentina | To analyze different community-based experiences in Buenos Aires, Argentina. Some focus specifically on drug use prevention, while others carry out activities we characterize as health promotion, including artistic, cultural, sports, and other initiatives. | Primary data collection; Evidence synthesis (i.e. review) |
| Contribution of Aboriginal Community-Controlled Health Services to improving Aboriginal health: an evidence review | Campbell 2018 | Australia | To gather and examine the available evidence that highlights the role of Aboriginal Community Controlled Health Services in enhancing the health of the Aboriginal community. | Analysis of existing data (secondary analysis); Evidence synthesis (i.e. review) |
| Social capital, social movements and global public health: Fighting for health-enabling contexts in marginalised settings. | Campbell 2020 | South Africa, UK | To conduct a literature review on the role of community mobilization and social capital in global public health promotion. | Analysis of existing data (secondary analysis); Evidence synthesis (i.e., review) |
| La prevencion de los consumos problematicos de drogas desde una perspectiva comunitaria: un modelo para armar | Capriati 2015 | Argentina | To identify institutions and organizations aligned with the socio-community model for addressing problematic drug use, with the aim of expanding and consolidating this approach. | Primary data collection |
| [A governance approach applied to analysing research into unemployed workers in the city of Medellin in Colombia]. | Cardona 2010 | Colombia | To apply Marc Hufty’s governance framework to analyze actor interactions in a research-intervention project on the health coverage of unemployed TC workers in Medellín (2004–2007). | Primary data collection |
| Tackling health inequalities and social exclusion through partnership and community engagement? A reality check for policy and practice aspirations from a Social Inclusion Partnership in Scotland | Carlisle 2010 | Scotland | To describe the complexity of operationalizing a participatory initiative addressing health inequalities in a community facing disadvantage. | Primary data collection |
| Barreras, oportunidades y tacticas para participar en salud segun asociaciones de usuarios del valle del cauca, colombia | Castañeda Gamboa 2015 | Colombia | To identify and describe perceived barriers and opportunities for participation in the General Social Security Health System, and to analyze the strategies developed by user associations in Valle del Cauca from a constructivist perspective. | Analysis of existing data (secondary analysis) |
| Multisectoral partnerships to tackle complex health issues at the community level: lessons from a Healthy Communities Approach in rural Alberta, Canada | Chaisson 2022 | Canada | To highlight the findings of an evaluation of the Alberta Healthy Communities Approach. | Primary data collection |
| Promoting Sustainable Community Change in Support of Older Adult Physical Activity: Evaluation Findings from the Southeast Seattle Senior Physical Activity Network (SESPAN) | Cheadle 2009 | USA (Seattle) | To evaluate SESPAN (Southeast Senior Physical Activity Network), including implementation-related lessons, and inform the improvement of processes of preventive services delivery. | Primary data collection; Analysis of existing data |
| Project CHARGE: Building an Urban Health Policy Advocacy Community | Chin 2012 | USA | To discuss the strategies used by the Coalition for Health Access to Reach Greater Equity (Project CHARGE) for building a successful coalition. | Primary data collection |
| Alliance System and Policy Change: Necessary Ingredients for Improvement in Diabetes Care and Reduction of Disparities | Clark 2014 | USA | To identify and understand the system and policy changes that were implemented to reduce diabetes disparities among vulnerable populations. Specifically, the researchers focused on the efforts of the Alliance to Reduce Disparities in Diabetes, which targeted low-income African American, Native American, and Latino communities. | Primary data collection |
| Understanding similarities in the local implementation of a healthy environment programme: Insights from policy studies | Clavier 2012 | Canada | To evaluate how a procedural public health program, focused on creating healthy environments (HE) for vulnerable families in Quebec, Canada, is implemented at the local level, including a focus on how funding for local projects is distributed and utilized. | Primary data collection; Analysis of existing data (secondary analysis) |
| Enabling volunteer health planning capacity: a rural Canadian case study | Coady 2007 | Canada | To identify factors (specifically, any resources, capacity building supports and opportunities) which bolster the capacity of volunteers to participate in local health planning, using the case of community health board volunteers in the Guysborough Antigonish Strait Health Authority. | Primary data collection |
| Community health assessment of the programme "Barcelona Health in the Neighbourhoods". Methodology for a participatory process | Colell 2018 | Spain | To present the methodology used in a participatory health diagnosis conducted in an urban context, based on the experience of the Barcelona Salut als Barris (BSaB) program. | Primary data collection; Analysis of existing data (secondary analysis) |
| The Untold Story: Examining Ontario's Community Health Centres' Initiatives to Address Upstream Determinants of Health | Collins 2014 | Canada | To investigate the following characteristics in certain Ontario Community Initiatives: Scope thereof, resources needed, partnerships, challenges and successes. | Primary data collection |
| Integrating research and action: a systematic review of community-based participatory research to address health disparities in environmental and occupational health in the USA | Cook 2008 | USA | To investigate the degree to which community-based participatory research involves community-level action and to identify the factors that facilitate such action. | Evidence synthesis (i.e. review) |
| Community knowledge in environmental health science: co-producing policy expertise | Corburn 2007 | USA | To show how involving the public in science policy can lead to policies that are both based on good science and reflect the needs of the people they affect. The research explores the advantages and disadvantages of using community-based knowledge when making decisions about environmental health. | Primary data collection; Analysis of existing data (secondary analysis) |
| Exploring the role of community engagement in improving the health of disadvantaged populations: a systematic review. | Cyril 2015 | Africa, Bangladesh, China, India, Iran, UK, Canada | To investigate the effectiveness of community engagement (CE) models in improving health outcomes in disadvantaged populations. The authors conducted a systematic review of existing research on the topic and found that CE can be an effective way to improve health behaviors, public health planning, health service access, health literacy, and other health outcomes. | Analysis of existing data (secondary analysis); Evidence synthesis (i.e. review) |
| Service user engagement in health service reconfiguration: a rapid evidence synthesis | Dalton 2015 | UK | To synthesize evidence on effective community (specifically, public and patient) engagement in the process of reconfiguring health services. | Evidence synthesis (i.e. review) |
| Improving social accountability processes in the health sector in sub-Saharan Africa: a systematic review. | Danhoundo 2018 | Sub-Saharan Africa | To determine which condition(s) enable effective social accountability in the sub-Saharan Africa context. | Evidence synthesis (i.e. review) |
| Community co-produced mental health initiatives in rural Australia: A scoping review | DeCotta 2021 | Australia | To identify the defining characteristics of rural mental health initiatives co-produced with communities in Australia. | Evidence synthesis (i.e. review) |
| Inclusive public participation in health: Policy, practice and theoretical contributions to promote the involvement of marginalised groups in healthcare | deFreitas 2015 | The Netherlands | To identify the enablers of participation by marginalized groups, conceptualized using the Participation Chain Model (PCM) and based on a Dutch case study of user participation in mental health advocacy. | Primary data collection; Analysis of existing data (secondary analysis) |
| Searching for new community engagement approaches in the Netherlands: a realist qualitative study | DeWeger 2020 | The Netherlands | To describe understanding and operationalization of community engagement in the healthcare system. | Primary data collection |
| Salut als Barris en Barcelona, una intervencion comunitaria para reducir las desigualdades sociales en salud | Diez David 2022 | Spain | To describe the experience and challenges of the Salut als Barris program in Barcelona. | Analysis of existing data (secondary analysis) |
| Implementation of the Healthy Cities' principlesand strategies: an evaluation of the Israel Healthy Cities Network | Donchin 2006 | Israel | To evaluate the extent to which 'Healthy Cities' principles and strategies have been implemented in each network city and to assess each network's contributions to its member cities. | Primary data collection |
| A qualitative analysis of vaccine decision makers' conceptualization and fostering of 'community engagement' in India | Dutta 2020 | India | To understand how community engagement was approached during the Decade of Vaccines, between 2010 and 2020, within India. | Primary data collection; Analysis of existing data (secondary analysis) |
| Protestas sociales por la salud en Colombia: la lucha por el derecho fundamental a la salud, 1994-2010 | Echeverry-López 2015 | Colombia | To reconstruct the transgressive struggle for health in Colombia during the implementation of health reform (1994–2010), focusing on social protests led by subaltern actors. | Analysis of existing data (secondary analysis) |
| Mobilizing communities to improve maternal health: results of an intervention in rural Zambia | Ensor 2014 | Zambia | To evaluate the impact of a complex community intervention implemented in rural Zambia to improve maternal health. | Primary data collection |
| Academic practice–policy partnerships for health promotion research: Experiences from three research programs | Eriksson 2014 | Sweden | To explore factors that foster academic practice policy partnerships, and to systematically review three projects with the aim of understanding the dynamic context in which the researcher-practitioner-policymaker-community relationship unfolds. | Primary data collection; Analysis of existing data |
| Community participation in a rural community health trust: the case of Lawrence, New Zealand | Eyre 2003 | New Zealand | To assess how well a community health trust model functioned in terms of community participation. The researchers used a framework called the "pentagram model" to analyze the Lawrence trust, a rural health trust in New Zealand. This model looks at five aspects of community participation:  (1) Resource mobilization: How well did the community raise and manage funds for the trust?  (2) Needs assessment: Did the trust consider the health needs of the entire community?  (3) Management: How were decisions about the trust made?  (4) Organization: How was the trust structured?  (5) Leadership: Who were the leaders in the trust, and how did they represent the community? | Analysis of existing data (secondary analysis)  Primary data collection |
| Urban primary health care in Africa: a comparative analysis of city-wide public sector projects in Lusaka and Dar es Salaam. | Few 2003 | Tanzania, Zambia | To compare two projects intended to enhance public urban primary health care by enhancing the poor's access to quality health services and promoting community participation in health. | Primary data collection; Analysis of existing data (secondary analysis) |
| Missing knowledge of gendered power relations among non-governmental organisations doing right to health work: a case study from South Africa | FontesMarx 2018 | South Africa | To explore the extent and impacts of gender awareness and inclusiveness in South African organizations advocating for the right to health. | Primary data collection |
| La Revolucion Ciudadana and social medicine: Undermining community in the state provision of health care in Ecuador. | Friederic 2019 | Ecuador | To examine the impact of Ecuador's state-centric health reforms on community participation and community organizing, focusing on the evolved relationships between civil society (local and translation) and the state via the case of Las Colinas. | Primary data collection |
| Translating Workforce Development Policy Interventions for Community Health Workers: Application of a Policy Research Continuum | Fulmer 2020 | USA | To describe a research method for evaluating the effectiveness of public health policies, namely the policy research continuum, and apply this method to evaluate the development of the community health worker (CHW) workforce. | Analysis of existing data (secondary analysis) |
| Dissonances and disconnects: the life and times of community-based accountability in the National Rural Health Mission in Tamil Nadu, India | Gaitonde 2020 | India | To document the process of implementing the Community Action for Health Project (CAH) in Tamil Nadu, specifically the institutional processes involved. | Primary data collection |
| Expanding the Debate: Citizen Participation for the Implementation of the Right to Health in Brazil. | Garcia 2018 | Brazil | To explore the potential of Brazil's National Health Council to cultivate accountability by way of citizen participation in health planning. | Primary data collection |
| Anchoring contextual analysis in health policy and systems research: A narrative review of contextual factors influencing health committees in low and middle income countries | George 2015 | Global | To examine the contextual factors that influence health committees (HCs) and their ability to function effectively. The authors reviewed literature on HCs published after 1996 and identified four key contextual spheres that influence HCs: community, health facility, health administration, and society. | Evidence synthesis (i.e. review) |
| The equity impacts of community financing activities in three African countries. | Gilson 2000 | Benin, Kenya, Zambia | To investigate the impacts (related to equity) of community financing initiatives in Benin, Kenya and Zambia, alongside the mechanism(s) by which impacts were made | Primary data collection; Analysis of existing data (secondary analysis) |
| Health facility committees and facility management - exploring the nature and depth of their roles in Coast Province, Kenya | Goodman 2011 | Kenya | To investigate the effectiveness of Health Facility Committees as a means for improving community accountability in healthcare delivery within Kenya. | Primary data collection |
| Implementing large-scale programmes to optimise the health workforce in low- and middle-income settings: a multicountry case study synthesis | Gopinathan 2014 | Brazil, Ethiopia, India, Iran, Malawi, Venezuela, Zimbabwe | To discern the factors which shape implementation of large programs aiming to optimize the health workforce. | Evidence synthesis (i.e. review) |
| A community-led mobile health clinic to improve structural and social determinants of health among (im)migrant workers | Guillot-Wright 2022 | USA | To understand the impact of a community-led mobile health clinic co-designed with seafood workers. | Primary data collection |
| Nepal's Health Facility Operation and Management Committees: exploring community participation and influence in the Dang district's primary care clinics | Gurung 2018 | Nepal | To characterize community representation within Health Facility Operation and Management Committees (HFMCs), and to determine the extent to which community representation in HFMCs influences decision-making processes. | Primary data collection; Analysis of existing data (secondary analysis) |
| Collaborative research and action to control the geographic placement of outdoor advertising of alcohol and tobacco products in Chicago | Hackbarth 2001 | USA | To evaluate the effectiveness of a 'voluntary code of principles' in constraining billboard advertisements for alcohol and tobacco products within Chicago. | Primary data collection |
| Community involvement in the development and implementation of chronic condition programmes across the continuum of care in high- and upper-middle income countries: A systematic review | Haldane 2020 | HIC, UMIC | To collate evidence surrounding community participation in programs for non-communicable disease management. | Evidence synthesis (i.e. review) |
| Service user and family participation in mental health policy making in Timor-Leste: a qualitative study with multiple stakeholders | Hall 2020 | Timor-Leste | To conduct a situational analysis of the participation of service users and their families in mental health policymaking, including assessment of challenges and enablers. | Primary data collection |
| Factors for success in mental health advocacy | Hann 2015 | Sierra Leone | To identify enablers of and barriers to mental health advocacy in a low-income country context, from the perspectives of mental health stakeholders. | Primary data collection |
| Advocacy, communication and social mobilisation for tuberculosis control in Pakistan: a qualitative case study | Haq 2013 | Pakistan | To investigate the National Tuberculosis Programme's (NTP's) advocacy, communication and social mobilization (ACSM) campaigns within Pakistan, namely:  (1) the mechanism(s) by which they engaged target populations;  (2) the extent to which they successfully promoted relevant services and behaviours;  (3) and the way(s) in which the ACSM campaigns could be improved. | Primary data collection |
| Advancing Indigenous primary health care policy in Alberta, Canada | Henderson 2018 | Canada | To describe a gathering which invited participants to explore and consider the use of PHC innovations from outside Alberta. | Primary data collection |
| Community-based directly observed treatment for TB patients to improve HIV services: a cross-sectional study in a South African province | Howell 2018 | South Africa | To describe the directly observed treatment (DOT) support provided by community health workers among tuberculosis patients in four municipalities in Free State province. | Primary data collection |
| Good governance and sustainability: a case study from Pakistan | Israr 2006 | Pakistan | To conduct a case study that exemplifies how good governance is crucial for sustaining donor-funded health systems projects in the public health sector. | Analysis of existing data (secondary analysis) |
| Can social accountability improve access to free public health care for the poor? Analysis of three Health Equity Fund configurations in Cambodia, 2015-17 | Jacobs 2020 | Cambodia | To describe the size of Health Equity Fund operations and their ability to enable poor people continued access to health care. | Primary data collection |
| State and non-state mental health service collaboration in a South African district: a mixed methods study | JansevanRensburg 2018 | South Africa | To explore the extent and nature of collaboration between state and non-state mental health services in the Mangaung Metropolitan District, Free State, South Africa. | Primary data collection |
| Role of innovative institutional structures in integrated governance. A case study of integrating health and nutrition programs in Chhattisgarh, India | Kalita 2012 | India | To highlight the importance of integrated governance in facilitating community participation, improving health service delivery, strengthening the accountability of public systems, and planning human resources. | Primary data collection; Analysis of existing data (secondary analysis) |
| Community participation in New Mexico's behavioral health care reform | Kano 2009 | USA | To examine how communities have responded to Local Collaboratives which are regional-level, state-designed community organizations addressing the behavioral health concerns of diverse cultural populations within New Mexico. | Primary data collection |
| Strengthening community leadership: evaluation findings from the california healthy cities and communities program | Kegler 2008 | USA | To examine how and the extent to which existing and emerging community leaders were involved as leaders in local projects, alongside the lessons learned from their involvement. | Primary data collection; Analysis of existing data (secondary analysis) |
| Community participation in rural health: a scoping review. | Kenny 2013 | "Developed" countries | To identify international-literature examples of community participation in higher level rural healthcare. | Evidence synthesis (i.e. review) |
| Community participation for rural health: a review of challenges. | Kenny 2015 | Universal | To outline evidence-informed considerations for the implementation of policies promoting community participation for health within rural contexts. | Evidence synthesis (i.e. review) |
| The functionality of health facility governing committees and their associated factors in selected primary health facilities implementing direct health facility financing in Tanzania: A mixed-method study | Kesale 2022 | Tanzania | To examine the functionality of Health Facility Governing Committees within Tanzanian primary health facilities, tasked with implementing fiscal decentralization through Direct Health Facility Financing. | Primary data collection |
| Public participation in decision-making on the coverage of new antivirals for hepatitis | Kieslich 2016 | Brazil, UK (England), South Korea and the USA | To explore how the public has participated in decisions regarding sofosbuvir access, which is a treatment for chronic hepatitis C, and particularly how public participation shaped the addressing of key considerations (e.g., equity). | Analysis of existing data (secondary analysis) |
| Operational challenges of engaging development partners in district health planning in Tanzania | Kiologwe 2022 | Tanzania | To build an understanding of the operational challenges of engaging development partners in district-level health planning within Tanzania. | Primary data collection |
| Barriers to the participation of people with psychosocial disability in mental health policy development in South Africa: a qualitative study of perspectives of policy makers, professionals, religious leaders and academics | Kleintjes 2013 | South Africa | To discuss the importance of including people with psychosocial disability in policy development related to mental health.The article argues that people with psychosocial disability have valuable insights to contribute to mental health policy, and that their exclusion from the policy development process is a detriment to the quality of those policies. The article also identifies several barriers that prevent people with psychosocial disability from participating in policy development, including stigma, poverty, and a lack of effective recovery and community supports. | Primary data collection |
| Towards an explanatory framework for national level maternal health policy agenda item evolution in Ghana: an embedded case study | Koduah 2018 | Ghana | To better understand decision-making processes which shape treatment of health policy agenda items. | Primary data collection |
| Undoing Racism Through Genesee County's REACH Infant Mortality Reduction Initiative | Kruger 2015 | USA | To assess the effectiveness of the Undoing Racism Workshop conducted by Genesee County Racial and Ethnic Approaches to Community Health Program, specifically its effectiveness in building an understanding of racism and how race/ethnicity-related issues influence maternal and infant health. | Primary data collection |
| Community action for health in India: evolution, lessons learnt and ways forward to achieve universal health coverage | Lahariya 2020 | India | To overview the participation of community-based and civil society organizations within policymaking in India over the previous two decades, describing three initiatives focused on strengthening such participation. | Analysis of existing data (secondary analysis) |
| Measures to strengthen primary health-care systems in low- and middle-income countries. | Langlois 2020 | Bangladesh, Cameroon, Colombia, Ethiopia, Georgia, Ghana, Indonesia, Kenya, Lebanon, Mexico, Mongolia, Nigeria, Pakistan, Peru, Rwanda, South Africa, Sri Lanka, Tanzania, Thailand, Uganda | To distill lessons for primary healthcare system strengthening in low- and middle-income countries. | Primary data collection |
| Improving health outcomes through community empowerment: A review of the literature | Laverack 2006 | Australia, Nepal, New Zealand, Polynesia, UK (England), USA | To define and elaborate on the concept of community empowerment, focusing on how relatively powerless individuals and groups can increase their control over factors affecting their lives and health, and to explore the influence of empowerment on well-being. | Analysis of existing data (secondary analysis) |
| Process evaluation of an academic-community-government partnership to reduce liver diseases attributable to hepatitis B virus | Le 2022 | USA | To provide a detailed evaluation and documentation of the goals, achievements, challenges, and future directions of the ACG partnership involved in addressing hepatitis B virus (HBV) disparities among racial/ethnic minority populations. The article specifically focuses on the activities and outcomes of the partnership during its first year. | Primary data collection; Analysis of existing data (secondary analysis) |
| "We come as friends": approaches to social accountability by health committees in Northern Malawi | Lodenstein 2019 | Malawi | To explore the role of Health Centre Advisory Committees (HCACs) in rural Malawi as interfaces for social accountability in the health system. Specifically, it investigates how HCACs facilitate interactions between citizens and health workers, handle complaints, and address poor health worker performance. | Primary data collection; Analysis of existing data (secondary analysis) |
| Managing community engagement initiatives in health and social care: lessons learned from Italy and the United Kingdom | Longo 2023 | Italy, UK | To characterize the features of diverse community interventions and inform the strengthening of both management and policy for greater health and social impact. | Primary data collection; Analysis of existing data |
| Working together to improve the mental health of indigenous children: A systematic review | Lopez-Carmen 2019 | Australia, Canada, New Zealand, Norway, USA | To examine the available literature surrounding intersectoral service integration processes and tools designed to improve mental healthcare for Indigenous children. | Evidence synthesis (i.e. review) |
| What is the evidence that the establishment or use of community accountability mechanisms and processes improves inclusive service delivery by governments, donors and NGOs to communities? | Lynch 2013 | Kenya, Nigeria, Sierra Leona, South Africa, Tanzania, Uganda | To pinpoint interventions that have demonstrated either positive or negative effects in enhancing community accountability and fostering inclusive service delivery. | Evidence synthesis (i.e. review) |
| Realising radical potential: building community power in primary health care through Participatory Action Research | Mabetha 2023 | South Africa | To analyze community power-building in primary healthcare through a participatory action research initiative within rural South Africa, and to explore how the process was designed to a sustainable PHC component. | Primary data collection |
| Feasibility, acceptability and initial outcome of implementing community scorecard to monitor community level public health facilities: experience from rural Bangladesh | Mahmood 2020 | Bangladesh | To outline the acceptability, feasibility, early outcomes and challenges of community scorecard implementation in community clinics. | Primary data collection; Analysis of existing data (secondary analysis) |
| Lessons in Community Health Activism: The Maternity Care Coalition, 1970-1990 | Maldonado 2014 | USA | To describe the history of the Care Coalition (MCC). By examining the MCC's past, the article explores how studying historical trends in family and community health can provide valuable understanding of current health issues. | Primary data collection; Analysis of existing data (secondary analysis); Other: Historical method |
| Decentralized health care priority-setting in Tanzania: evaluating against the accountability for reasonableness framework | Maluka 2010 | Tanzania | To describe and evaluate the health care priority setting process in Mbarali district, Tanzania. | Primary data collection |
| Decentralization and health care prioritization process in Tanzania: from national rhetoric to local reality | Maluka 2011 | Tanzania | To examines the processes, actors and contextual factors influencing decentralized health care priority setting. | Primary data collection; Analysis of existing data (secondary analysis) |
| From Concept to Application: The Impact of a Community-Wide Intervention to Improve the Delivery of Preventive Services to Children | Margolis 2001 | USA (North Carolina) | To evaluate the impacts of a community-wide intervention on preventive health service delivery for children. | Primary data collection; Analysis of existing data |
| Addressing rural and Indigenous health inequities in Canada through socially accountable health partnerships | Markham 2021 | Canada | To report on how the Partnership Pentagram Plus approach has been operationalized by First Nations Health Authority and British Columbia's Rural Coordination Centre. | Primary data collection |
| Chronic patient as intermittent partner for policy-makers: the case of patient participation in the fight against diabetes and HIV/AIDS in Mali | Martini 2019 | Mali | To explore the factors influencing patient participation in chronic disease policy making, using Mali as a case study. The article compares how patients with diabetes and HIV/AIDS participate in shaping health policy at the national level. | Primary data collection |
| Challenges to effective governance in a low income healthcare system: a qualitative study of stakeholder perceptions in Malawi | Masefield 2020 | Malawi | To explore health sector stakeholders' perceptions of the challenges to improving governance in Malawi's national health system within the post-2017 context of government attempts to articulate a way forward. | Primary data collection |
| Stakeholder engagement in the health policy process in a low income country: a qualitative study of stakeholder perceptions of the challenges to effective inclusion in Malawi | Masefield 2021 | Malawi | To explore the extent of stakeholder engagement in health policymaking from the perspectives of local stakeholders. | Primary data collection |
| Promoting community malaria control in rural Myanmar through an active community participation program using the participatory learning approach | Maung 2017 | Myanmar | To improve community engagement in malaria control efforts and raise awareness and knowledge of malaria prevention practices in rural Myanmar. This research was motivated by the high prevalence of malaria in Myanmar, affecting over 80% of townships, and the current top-down approach to malaria control implemented by the National Malaria Control Programme (NMCP). | Primary data collection |
| Implementing community participation through legislative reform: a study of the policy framework for community participation in the Western Cape province of South Africa | Meier 2012 | South Africa | To highlight generalizable best practices for developing policy in a manner that honours rights-based community participation. | Primary data collection; Evidence synthesis (i.e. review) |
| Established Tables and Emergent Huddles: Exploring the Processes of Participation Associated With the Policy Changes to Opioid Pharmacotherapy Treatment in Australia in the Context of COVID-19 | Mellor 2022 | Australia | To conduct a case study supporting the examination of interactions between emergent and established forms of community participation. | Primary data collection |
| “Sometimes it is difficult for us to stand up and change this”: an analysis of power within priority-setting for health following devolution in Kenya | Miller 2018 | Kenya | To conduct a power analysis focused on county-level prioirty-setting within Kenya. | Primary data collection; Analysis of existing data |
| Strengthening user participation through health sector reform in Colombia: a study of institutional change and social representation. | Mosquera 2001 | Colombia | To explore how different actors' social representations at various health system levels shape implementation of Colombia's participatory policy. |  |
| Community participation to improve health services for children: a methodology for a community dialogue intervention in Uganda | Muhwezi 2019 | Uganda | To evaluate the Community Dialogue strategies as part of the Community and District Empowerment for Scale-up project within Uganda, which aimed to enhance the uptake of health services for childhood diseases (diarrhea, pneumonia, and malaria) via community-based initatives and health systems strengthening. | Primary data collection |
| Tracking health sector priority setting processes and outcomes for human resources for health, five-years after political devolution: a county-level case study in Kenya | Munywoki 2020 | Kenya | To examine whether and how the health sector devolution within Kenya has strengthened priority setting practices and outcomes around human resources for health. | Primary data collection; Analysis of existing data (secondary analysis) |
| The impact of coalition characteristics on outcomes in community-based initiatives targeting the social determinants of health: a systematic review. | Nagorcka-Smith 2022 | The study does not explicitly focus on specific countries but rather includes research from a global perspective. It reviews studies from various geographic locations, although it does not specify a predominant focus on any single country or region. | To collate evidence illustrating the relationship between coalition characteristics and coalition outcomes, in the context of community-based initiatives targeting the social determinants of health. | Evidence synthesis (i.e. review) |
| Understanding the benefits and challenges of community engagement in the development of community mental health services for common mental disorders: Lessons from a case study in a rural South African subdistrict site | Petersen 2012 | South Africa | To explore the benefits and challenges of community participation in mental health services in low-resource settings, beyond scale-up of services. The authors argue that community participation can also lead to promoting culturally competent services and greater community control of mental health. | Primary data collection; Analysis of existing data (secondary analysis) |
| HIV prevention among gay and other men who have sex with men: public policy and social movements in Brazil, 1983-2019 | Pinheiro 2021 | Brazil | To reviews the history of HIV prevention among men who have sex with men, focusing on the influence of LGBTI+ and other social movements on HIV/AIDS policy within Brazil. | Primary data collection; Analysis of existing data |
| Expanding and improving urban outreach immunization in Patna, India | Pradhan 2012 | India | To describe and evaluate an urban outreach immunization intervention in Patna, India, intended to improve vaccination coverage amidst resourcing challenges. | Primary data collection |
| Community participation in local health boards in a decentralized setting: cases from the Philippines | Ramiro 2001 | Philippines | To examine the Local Health Board's role in facilitating broader community participation in health decision-making within the Philippines. | Primary data collection |
| Decentralization in India's health sector: insights from a capacity building intervention in Karnataka | RaoSeshadri 2019 | India | To report regarding and highlight lessons from the team's intervention implemented in Karnataka, India. | Primary data collection |
| Participation in Policy Discourse: New Form of Exclusion for Seniors with Disabilities? | Raymond 2013 | Canada | To conduct a critical discourse analysis of aging policy in Québec, Canada between 2005 and 2011. | Evidence synthesis (i.e. review) |
| Applying priority-setting frameworks: A review of public and vulnerable populations’ participation in health-system priority setting | Razavi 2019 | Multiple | To identify which stakeholders, including the public, have been involved in health system priority setting, with an emphasis on the participation of vulnerable populations. | Evidence synthesis (i.e. review) |
| Who is in and who is out? A qualitative analysis of stakeholder participation in priority setting for health in three districts in Uganda | Razavi 2019 | Uganda | To examine the participation of various stakeholders in priority setting processes, from the perspectives of district-level decision-makers. | Primary data collection |
| Critical Elements of Community Engagement to Address Disparities and Related Social Determinants of Health: The Centers of Disease Control and Prevention Community Approaches to Reducing Sexually Transmitted Disease Initiative | Rhodes 2020 | USA | To identify and examine the aspects of successful community engagement in the mitigation of sexually transmitted infections. | Primary data collection; Analysis of existing data (secondary analysis) |
| Participación social y comunitaria en  Atención Primaria de Salud - APS.  Bogotá: para la muestra un botón | Restrepo Vélez 2009 | Colombia | To identify, interpret, and analyze available evidence on community participation and empowerment experiences in primary health care in the Capital District of Colombia between 2004 and 2007. | Evidence synthesis (i.e. review) |
| A Public Health Learning Collaborative on Climate Change for Urban Health Departments, 2016-2018 | Rudolph 2020 | USA | To examine the efforts of urban local health departments to address climate change, equity, and health while weaving climate change considerations into program practice and participating in their localities' climate change initiatives. | Primary data collection; Analysis of existing data (secondary analysis) |
| Social exclusion and universal health coverage: health care rights and citizen-led accountability in Guatemala and Peru. | Samuel 2020 | Guatemela, Peru | To conduct two case studies, one in Guatemala and one in Peru, illustrating citizen-led accountability initiatives identifying and addressing the challenges of healthcare for groups facing social exclusion. |  |
| Los consejos de salud como instrumento de participacion comunitaria en La Rioja | Sainz-Ruiz 2019 | Spain | To describe the functioning and regulatory framework of the health councils of La Rioja, Spain, as citizen participation bodies in health governance. | Primary data collection; Analysis of existing data (secondary analysis) |
| Does Information and Communication Technology Add Value to Citizen-Led Accountability Initiatives in Health? Experiences from India and Guatemala. | Schaaf 2018 | Guatemala, India | To explore the contributions of Information and Communication Technology to efforts promoting government accountability for healthcare services in India and Guatemala. | Primary data collection; Analysis of existing data (secondary analysis) |
| The East Side Village Health Worker Partnership: integrating research with action to reduce health disparities | Schulz 2001 | USA (Detroit) | To examine the mechanisms through which research-practice partnerships between community residents and public health professionals can improve research methods, practice, and community-practitioner relationships. | Primary data collection |
| The Tribal Tobacco Education and Policy Initiative: Findings From a Collaborative, Participatory Evaluation | Scott 2017 | USA | To outline the emergent findings of an evaluation of the Tribal Tobacco Education and Policy initiative with four Tribal nations. | Analysis of existing data (secondary analysis) |
| Uptake of health insurance and the productive safety net program in rural Ethiopia | Shigute 2017 | Ethiopia | To assess the effects of the Community-Based Health Insurance (CBHI) scheme introduced in Ethiopia. The study primarily focuses on understanding how participation in the Productive Safety Net Program (PSNP), a social protection initiative, influences enrollment and dropout rates in the CBHI scheme. | Primary data collection; Analysis of existing data (secondary analysis) |
| Addressing Health Disparities from Within the Community: Community-Based Participatory Research and Community Health Worker Policy Initiatives Using a Gender-Based Approach | Simonsen 2017 | USA | To conduct a case study exemplifying the identification and tracking of as well as engagement with policy initiatives addressing obesity-related health disparities impacting women of colour in Utah, USA. | Primary data collection |
| Acceptability and trust of community health workers offering maternal and newborn health education in rural Uganda | Singh 2015 | Uganda | To investigate the challenges experienced by community health workers, specifically as they gain recognition in the community and begin the process of gaining community trust. | Primary data collection |
| Circus monkeys or change agents? Civil society advocacy for HIV/AIDS in adverse policy environments. | Spicer 2011 | Georgia, Kyrgyzstan, Ukraine | To discern the factors enabling and constraining civil society advocacy for HIV/AIDS- and drug-related policy reforms. | Primary data collection |
| Social transformation, collective health and community-based arts: 'Buen Vivir' and Ecuador's social circus programme. | Spiegel 2019 | Ecuador | To analyze a case of community-based arts being used to advance social transformation. | Primary data collection; Analysis of existing data (secondary analysis) |
| A qualitative exploration of stakeholder perceptions of the implementation of place-based working and its potential to reduce health inequality | Steer 2018 | UK (England) | To explore stakeholder perceptions of the implementation of a place-based work intervention to better understand underlying processes and contextual factors alongside the potential role of the intervention in reducing health inequality. | Primary data collection |
| Evaluation of the Southern Harm Reduction Coalition for HIV Prevention: Advocacy Accomplishments | Story 2018 | USA | To use the CCAT (community coalition action theory) lens to detail the mechanism(s) by which the Southern Harm Reduction Coalition achieved its accomplishments, particularly within a difficult community context. | Primary data collection |
| Achieving wider participation in strategic health planning: experience from the consultation phase of Liverpool's 'City Health Plan' | Strobl 2000 | UK | To evaluate the effect of the consultation in Liverpool on broad-based participation in City Health Plan development. | Primary data collection |
| Equity in access to maternal and child health services in five developing countries: what works. | Talukder 2010 | Bangladesh, Cambodia, Ghana, Pakistan, Tanzania | To investigate and evaluate various health service delivery models implemented in developing countries (specifically Bangladesh, Cambodia, Ghana, Pakistan, and Tanzania) with the aim of strengthening access to maternal and child health services. | Evidence synthesis (i.e. review) |
| The role of state and non-state actors in the policy process: the contribution of policy networks to the scale-up of antiretroviral therapy in Thailand | Tantivess 2008 | Thailand | To illustrate the extent to which public policymaking in Thailand is shifting toward the inclusion of civil society in the identification of health issues and the development of policies to address those. | Primary data collection; Analysis of existing data (secondary analysis) |
| Implementing community participation via interdisciplinary teams in primary care: An Irish case study in practice | Tierney 2018 | Ireland | To analyze the operationalization of community participation in the context of primary care teams within Ireland. | Primary data collection |
| "We are everything to everyone": a systematic review of factors influencing the accountability relationships of Aboriginal and Torres Strait Islander health workers (AHWs) in the Australian health system | Topp 2018 | Australia | To synthesize evidence regarding Aboriginal Health Workers' accountability experiences in Australia's health system | Evidence synthesis (i.e. review) |
| Engaging Youth in Food Activism in New York City: Lessons Learned from a Youth Organization, Health Department, and University Partnership | Tsui 2012 | USA | To evaluate and discuss the implementation and outcomes of the Health Equity Project, a community-based intervention launched by the New York City Department of Health and Mental Hygiene. | Primary data collection |
| Exploring operational barriers encountered by community midwives when delivering services in two provinces of Pakistan: A qualitative study | Ur Rehman 2015 | Pakistan | To discern (1) the barriers which community midwives face during service delivery, from their perspectives and the perspectives of their manager and (2) any other factors which relate to poor performance of community midwives in maternal, neonatal and child health service delivery. | Primary data collection |
| APS y acceso universal a los servicios de salud en las condiciones del SGSSS de Colombia. El caso `salud a su hogar´ en Bogotá | Vega Romero 2006 | Colombia | To describe the opportunities and challenges faced by policymakers at the Bogotá District Health Secretariat during the 2004–2005 implementation of the Comprehensive Primary Health Care strategy “Salud a Su Hogar,” including operational changes in health services and preliminary outcomes. | Primary data collection; Analysis of existing data (secondary analysis) |
| Designing for action: adapting and implementing a community-based newborn care package to affect national change in Uganda | Waiswa 2015 | Uganda | To detail the process of designing an evidence-informed, community-based package focused on maternal and newborn care in rural eastern Uganda, and to discern the mechanism(s) used for advocacy and dissemination for policy change and uptake nationally. | Primary data collection; Analysis of existing data (secondary analysis) |
| Cocreating evidence-informed health equity policy with community | Walker 2022 | USA | To examine the feasibility of a rapid process for prioritizing policy options focused on health equity and informed by community voices, research evidence, and broader public health priorities. | Primary data collection; Analysis of existing data (secondary analysis); Evidence synthesis (i.e. review) |
| Together Helping Reduce Youth Violence for Equity (ThrYve): Examining the Development of a Comprehensive Multisectoral Approach to Youth Violence Prevention | Watson-Thompson 2020 | USA | To examine the implementation of the Institute of Medicine's Model for Collaborative Action in Communities to support the development of ThrYve (Together Helping Reduce Youth Violence for Equity). | Primary data collection; Analysis of existing data (secondary analysis) |
| Applying a cervical cancer control model in Soacha, Colombia | Wiesner-Ceballos 2008 | Colombia | To conduct a study in a Colombian municipality with a high concentration of uninsured or subsidized population, applying a cancer control model based on three levels of action and social strategies from a rights-based approach. | Primary data collection |
| Community perceptions of universal health coverage in eight districts of the Northern and Volta regions of Ghana | Wright 2020 | Ghana | To evaluate the Community-Based Health Planning and Services (CHPS) program in Ghana from the perspectives of women, adolescent girls, and community leaders. | Primary data collection; Analysis of existing data (secondary analysis) |
| Engaging Consumers in Medicaid Program Design: Strategies from the States | Zhu 2021 | USA | To investigate and understand consumer engagement in the design and implementation of state Medicaid programs. | Primary data collection |
| Increased fairness in priority setting processes within the health sector: the case of Kapiri-Mposhi District, Zambia | Zulu 2014 | Zambia | To outline the local-level practices and perceptions of fair priority setting alongside how these practices and perceptions evolved due to an Accountability for Reasonableness intervention. | Primary data collection; Analysis of existing data (secondary analysis) |
| Defining Democracy and the Terms of Engagement with the Postsocialist Polish State: Insights from HIV/AIDS | Owczarzak 2009 | Poland | To explore the shifting boundaries of state responsibility from Poland's socialist period through the postsocialist transition using the case of HIV/AIDS and the debates that surrounded it. | Primary data collection |

**References**

Haldane, Victoria, Fiona LH Chuah, Aastha Srivastava, Shweta R. Singh, Gerald CH Koh, Chia Kee Seng, and Helena Legido-Quigley. 2019. “Community Participation in Health Services Development, Implementation, and Evaluation: A Systematic Review of Empowerment, Health, Community, and Process Outcomes.” *PloS One* 14(5):e0216112.

Hebbar, Pragati Bhaskar, Vivek Dsouza, Upendra Bhojani, Nuggehalli Srinivas Prashanth, Onno CP van Schayck, Giridhara R. Babu, and Gera E. Nagelhout. 2022. “How Do Tobacco Control Policies Work in Low-Income and Middle-Income Countries? A Realist Synthesis.” *BMJ Global Health* 7(11):e008859. doi:10.1136/bmjgh-2022-008859.

Pawson, Ray, Trisha Greenhalgh, Gill Harvey, and Kieran Walshe. 2005. “Realist Review-a New Method of Systematic Review Designed for Complex Policy Interventions.” *Journal of Health Services Research & Policy* 10(1_suppl):21–34.

Wong, Geoff, Trish Greenhalgh, Gill Westhorp, Jeanette Buckingham, and Ray Pawson. 2013. “RAMESES Publication Standards: Realist Syntheses.” *BMC Medicine* 11:21. doi:10.1186/1741-7015-11-21.
